# Tumour suppressor death-associated protein kinase targets cytoplasmic HIF-1α for Th17 suppression

**DOI:** 10.1038/ncomms11904

**Published:** 2016-06-17

**Authors:** Ting-Fang Chou, Ya-Ting Chuang, Wan-Chen Hsieh, Pei-Yun Chang, Hsin-Yu Liu, Shu-Ting Mo, Tzu-Sheng Hsu, Shi-Chuen Miaw, Ruey-Hwa Chen, Adi Kimchi, Ming-Zong Lai

**Affiliations:** 1 Graduate Institute of Life Sciences, National Defense Medical College, Taipei 11490, Taiwan; 2 Institute of Molecular Biology, Academia Sinica, Taipei 11529, Taiwan; 3 Department of Medical Research, National Taiwan University Hospital, Taipei 10002, Taiwan; 4 Institute of Immunology, National Taiwan University, Taipei 10057, Taiwan; 5 Institute of Biological Chemistry, Academia Sinica, Taipei 11529, Taiwan; 6 Institute of Molecular Medicine, National Taiwan University, Taipei 10057, Taiwan; 7 Department of Molecular Genetics, Weizmann Institute of Science, Rehovot 76100, Israel

## Abstract

Death-associated protein kinase (DAPK) is a tumour suppressor. Here we show that DAPK also inhibits T helper 17 (Th17) and prevents Th17-mediated pathology in a mouse model of autoimmunity. We demonstrate that DAPK specifically downregulates hypoxia-inducible factor 1α (HIF-1α). In contrast to the predominant nuclear localization of HIF-1α in many cell types, HIF-1α is located in both the cytoplasm and nucleus in T cells, allowing for a cytosolic DAPK–HIF-1α interaction. DAPK also binds prolyl hydroxylase domain protein 2 (PHD2) and increases HIF-1α-PHD2 association. DAPK thereby promotes the proline hydroxylation and proteasome degradation of HIF-1α. Consequently, DAPK deficiency leads to excess HIF-1α accumulation, enhanced IL-17 expression and exacerbated experimental autoimmune encephalomyelitis. Additional knockout of HIF-1α restores the normal differentiation of *Dapk*^*−/−*^ Th17 cells and prevents experimental autoimmune encephalomyelitis development. Our results reveal a mechanism involving DAPK-mediated degradation of cytoplasmic HIF-1α, and suggest that raising DAPK levels could be used for treatment of Th17-associated inflammatory diseases.

## Abstract (web summary)

HIF-1α is critical for Th17 differentiation. Here the authors show that DAPK (Death-Associated Protein Kinase) inhibits Th17 differentiation and immunopathology in a mouse model of multiple sclerosis by promoting HIF1-α binding to its negative regulator PHD2.

## Graphical abstract

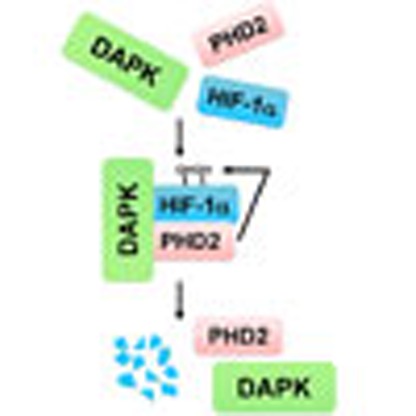

Upon activation, the T helper 17 (Th17) subset of immune cells plays critical roles in modulating tissue inflammation and combating microbial infections. However, due to their inflammatory nature, Th17 cells also contribute to autoimmune diseases^1^,^2^,^3^. Experimental autoimmune encephalomyelitis (EAE) is a well-studied mouse model for multiple sclerosis that is also mediated by Th17 (refs 4, 5, 6). Th17 cells differ from the Th1 and Th2 lineages in secretion of interleukin (IL)-17 (refs 7, 8), which induces inflammatory gene expression in target cells and leads to pathogenesis in the EAE model^9^. Transforming growth factor (TGF)-β is critical for the commitment to the Th17 lineage^10^,^11^. TGF-β acts synergistically with the STAT3-activating cytokines, IL-6, IL-21 and IL-23, to promote RORγt expression and Th17 differentiation^4^,^7^,^12^,^13^,^14^,^15^,^16^. The Th17-specific transcription factor RORγt^12^ acts together with RORα and STAT3 (ref. 17) to induce full Th17 cell differentiation.

Hypoxia-inducible factor-1α (HIF-1α) is an oxygen tension sensor widely expressed in different cell types, including Th17 cells. In the presence of O_2_, HIF-1α is hydroxylated at Pro402 and Pro564 by prolyl hydroxylase domain protein 2 (PHD2)/PHD3, followed by ubiquitination by the von Hippel–Lindau (VHL)-containing E3 complex that promotes proteasome degradation^18^,^19^,^20^,^21^,^22^. At low oxygen tension, HIF-1α is stabilized by inactivation of PHD2/PHD3 (refs 18, 19, 20, 21, 22). Once stabilized, HIF-1α activates the expression of target genes involved in hypoxic responses. HIF-1α is also upregulated by inflammatory cytokines in normoxic conditions^23^.

The *Hif1a* transcript is constitutively expressed in T lymphocytes, and the HIF-1α protein is detected after T-cell receptor (TCR) stimulation under hypoxic conditions^24^,^25^. HIF-1α is highly expressed in Th17 cells^26^,^27^, priming at physiological oxygen tension in the presence of inflammatory cytokines. HIF-1α plays a prominent role in Th17 cell differentiation^26^,^27^ by activating the transcription of *Rorc* (RORγt), and it helps recruit CBP/p300 to the RORγt transcription complex but does not directly bind to the IL-17 promoter^27^. Additionally, HIF-1α increases glycolysis by inducing the expression of glycolytic enzymes, which further contributes to Th17 development^26^,^28^. HIF-1α also contributes to the survival of Th17 cells by coordination with Notch to enhance Bcl-2 expression^29^. In contrast, targeted degradation of HIF-1α by miR-210 negatively regulates Th17 differentiation^30^.

HIF-1α promotes carcinogenesis and is a prominent cancer target^18^,^19^. Various HIF-1α inhibitors have been identified and are currently being studied for their efficacy in cancer therapy^18^,^19^,^31^,^32^. Presumably, HIF-1α inhibitors could also be used for treatment of Th17-mediated inflammatory diseases. However, HIF-1α is essential for oxygen homoeostasis, and curtailment of the protective effects of HIF-1α by HIF-1α inhibitors may limit their application.

Death-associated protein kinase (DAPk/DAPK) is a multi-domain serine/threonine kinase regulated by calcium^33^,^34^. DAPK belongs to the DAPK family, which also contains DAPK-related protein 1 and zipper-interacting protein kinase (also called DAPK3), both of which share 80% identity in their kinase domains with DAPK^33^. The DAPK family also contains two distantly related kinases: DAPK-related apoptosis inducing kinase 1 and 2 (DRK1 and DRK2)^35^. DAPK family members are pro-apoptotic proteins and function as tumour suppressors, and are specifically downregulated in many types of cancer^36^,^37^,^38^,^39^,^40^,^41^. DAPK participates in a wide variety of cellular events—including apoptosis, autophagy, membrane blebbing and stress fibre formation—that contribute to its tumour suppressor functions. In T lymphocytes, DAPK inhibits T-cell activation by suppressing TCR-induced nuclear factor (NF)-κB activation^42^. DAPK is induced by TGF-β (ref. 43), and is present in the early precursors of Th17, but the role of DAPK in Th17 immune cells is unclear.

In the present study, we found that DAPK negatively regulates Th17 differentiation. DAPK deficiency leads to preferential Th17 differentiation and exacerbated EAE induction. During the differentiation of Th17, the presence of DAPK is accompanied by downregulation of HIF-1α. We further found that, in contrast to the exclusive nuclear localization of HIF-1α in most other cells, HIF-1α is located in both the cytoplasm and nucleus of T cells, allowing the cytosolic binding of DAPK and subsequent HIF-1α degradation. Our results illustrate a novel mechanism of Th17 regulation by downregulating cytoplasmic HIF-1α, and suggest the therapeutic potential of increasing DAPK levels in Th17-mediated inflammatory diseases.

## Results

### DAPK inhibits T-cell activation and attenuates EAE

Our previous studies showed that expression of the dominant negative mutant of DAPK, [K42A]DAPK, leads to increased IL-2 production and NF-κB activation in T-cell lines^42^. Extending these studies, we examined T cells from the *Dapk*^−/−^ mouse^44^. T-cell development was not affected by DAPK deficiency, as illustrated by the normal thymic and peripheral T-cell populations in the *Dapk*^−/−^ mouse (Supplementary Fig. 1a,b). Following TCR stimulation, *Dapk*^−/−^ T-cell proliferation was enhanced relative to the wild-type (WT) T cells (Supplementary Fig. 2a). This was associated with increased IL-2 and interferon (IFN)-γ production in naive *Dapk*^−/−^ T cells (Supplementary Fig. 2b). An increase in IL-17 was also observed in *Dapk*^−/−^ T cells (Supplementary Fig. 2c). In contrast, the production of IL-4 in primary T cells was not affected by DAPK deficiency (Supplementary Fig. 2c). Additionally, IFN-γ production was similar between WT and *Dapk*^−/−^ T cells when cultured in Th0 conditions (Supplementary Fig. 2d). A prominent increase in NF-κB activation, indicated by the enhanced nuclear translocation of p65 (RelA), was also observed in *Dapk*^−/−^ T cells stimulated through the TCR (Supplementary Fig. 2e).

**Figure 1:**
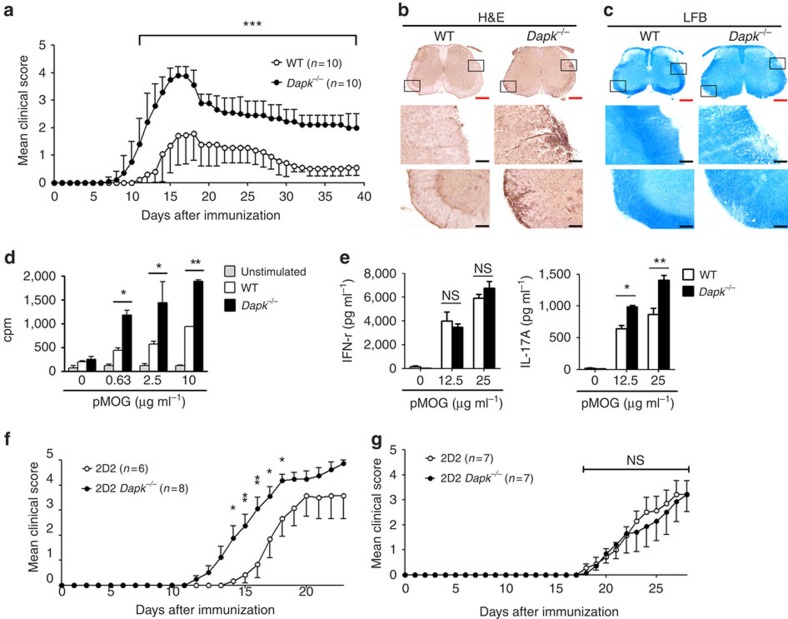
DAPK deficiency increases T-cell activation and EAE exacerbation. (**a**) EAE induction in wild-type (WT) and *Dapk*^*−/−*^ mice. DAPK knockout and WT control mice were immunized with 200 μg of myelin oligodendrocyte glycoprotein (MOG) peptide 35–55 emulsified in CFA, followed by intraperitoneal injection of 200 ng pertussis toxin at day 0 and day 2. The progression of disease was monitored. Values are mean±s.d. *n*=10 for each group. (**b**,**c**) Increased mononuclear cell infiltration and demyelination in spinal cords from primed *Dapk*^*−/−*^ mice. Spinal cords from WT and *Dapk*^*−/−*^ mice were isolated 11 days after MOG immunization, and fixed and frozen sections were obtained. Tissue sections were stained with hematoxylin and eosin (H&E) (**b**) and luxol fast blue (LFB) (**c**). Red scale bar, 400 μm; black scale bar, 80 μm. Photos are representative of three mice in each group. (**d**) Enhanced T-cell response to MOG (33–55) in *Dapk*^*−/−*^ mice. Splenic CD4 T cells were harvested 9 days after immunization. Cells were stimulated with irradiated autologous presenting cells plus MOG peptide, and the incorporation of thymidine was determined 72 h later. (**e**) Increased IL-17 production due to MOG (33–55) stimulation in *Dapk*^*−/−*^ T cells. The secretion of IFN-γ and IL-17 was quantitated in T cells from **d**. Data (**d**,**e**) are mean±s.d. (*n*=3), and are representative of three independent experiments. (**f**) 2D2 *Dapk*^*−/−*^ Th17 cells induced exacerbated EAE in *Rag1*^*−/−*^ mice. CD4^+^ T cells from 2D2 or 2D2 *Dapk*^*−/−*^ mice were differentiated into Th17 cells *in vitro*. 2D2 or 2D2 *Dapk*^*−/−*^ Th17 cells were re-stimulated and transferred intravenously to *Rag1*^*−/−*^ mice, followed by intraperitoneal administration of pertussis toxin. Mice were monitored for clinical signs of paralysis and recorded daily. (**g**) Comparable EAE generation by 2D2 WT Th1 cells and 2D2 *Dapk*^*−/−*^ Th1 cells in *Rag1*^*−/−*^ mice. 2D2 or 2D2 *Dapk*^*−/−*^ Th1 cells were transferred to *Rag1*^*−/−*^ mice, and clinical signs were monitored, as described in **f**. Values are mean±s.e.m. (**f**,**g**). *P<0.05, **P<0.01, ***P<0.001 for unpaired *t*-test. NS, not significant.

We further examined whether the enhanced T-cell activation in *Dapk*^−/−^ mice led to increased susceptibility to autoimmune diseases, using EAE as our model. After priming with myelin oligodendrocyte glycoprotein (MOG) peptide, disease onset was earlier in *Dapk*^−/−^ mice than their WT littermates (Fig. 1a). Additionally, the severity of encephalomyelitis was higher in *Dapk*^−/−^ mice than their WT counterparts. This was correlated with an increase in mononuclear cell infiltration and demyelination in spinal cords from primed *Dapk*^−/−^ mice (Fig. 1b,c). We also isolated T cells from the primed mice and determined their reactivity towards antigen. Consistent with exacerbated EAE generation, the response to MOG peptide in *Dapk*^−/−^ T cells was nearly double that of control T cells (Fig. 1d).

We further used transgenic mice with T-cell-specific expression of [ΔCAM]DAPK^42^, the constitutively active form of DAPK, to analyse EAE induction. T-cell development was normal in [ΔCAM]DAPK-transgenic mice (Supplementary Fig. 3a,b). We increased the dose of MOG peptide for a more profound EAE response in the control mice, allowing us to differentiate any inhibitory effect from the DAPK transgene. The expression of [ΔCAM]DAPK in T cells suppressed EAE generation (Supplementary Fig. 3c). The EAE symptoms were attenuated in [ΔCAM]DAPK-transgenic mice in comparison with WT littermate controls. Reduced responses to MOG peptide were also found for T cells isolated from [ΔCAM]DAPK-transgenic mice (Supplementary Fig. 3d). Together, these results demonstrate that DAPK deficiency enhanced EAE generation, whereas T-cell-specific expression of active DAPK suppressed EAE induction, suggesting that DAPK in T cells negatively regulates EAE pathogenesis.

Both Th17 and Th1 contribute to the development of EAE and multiple sclerosis^6^. Notably, IL-17 production, but not IFN-γ generation, was increased in MOG-primed *Dapk*^−/−^ T cells (Fig. 1e), suggesting a preferential expansion of DAPK-deficient Th17 cells. We examined the expression of IFN-γ and IL-17 in mononuclear cells infiltrated into the spinal cords of EAE-induced mice. During the peak of EAE induction, IFN-γ^+^ cells were more abundant than IL-17^+^ cells in spinal cords from WT mice (Supplementary Fig. 4a). Conversely, more IL-17^+^ cells than IFN-γ^+^ cells were detected in the spinal cords from *Dapk*^−/−^ mice with encephalomyelitis (Supplementary Fig. 4a). Quantitation shows an increased IL-17^+^:IFN-γ^+^ cells ratio in EAE-inducing *Dapk*^−/−^ mice (Supplementary Fig. 4b).

We further examined the induction of EAE in *Rag1*^*−/−*^ mice through adoptive transfer of 2D2 T cells. The adoptive transfer of MOG-specific 2D2 cells that were differentiated into Th17 cells allows for assessment of the effect of DAPK on the induction of antigen-specific and Th17-specific EAE. *Dapk*^−/−^ 2D2 Th17 cells triggered earlier disease onset and induced more severe encephalomyelitis than WT 2D2 Th17 cells (Fig. 1f). In contrast, the EAE onset and disease severity induced by *Dapk*^−/−^ 2D2 Th1 cells and WT 2D2 Th1 cells were similar (Fig. 1g). Together, these results suggest that DAPK deficiency leads to an increase in encephalitogenic Th17 cells, but not encephalitogenic Th1 cells.

### Preferential Th17 differentiation from *Dapk* ^*−/−*^ T cells

**Figure 2:**
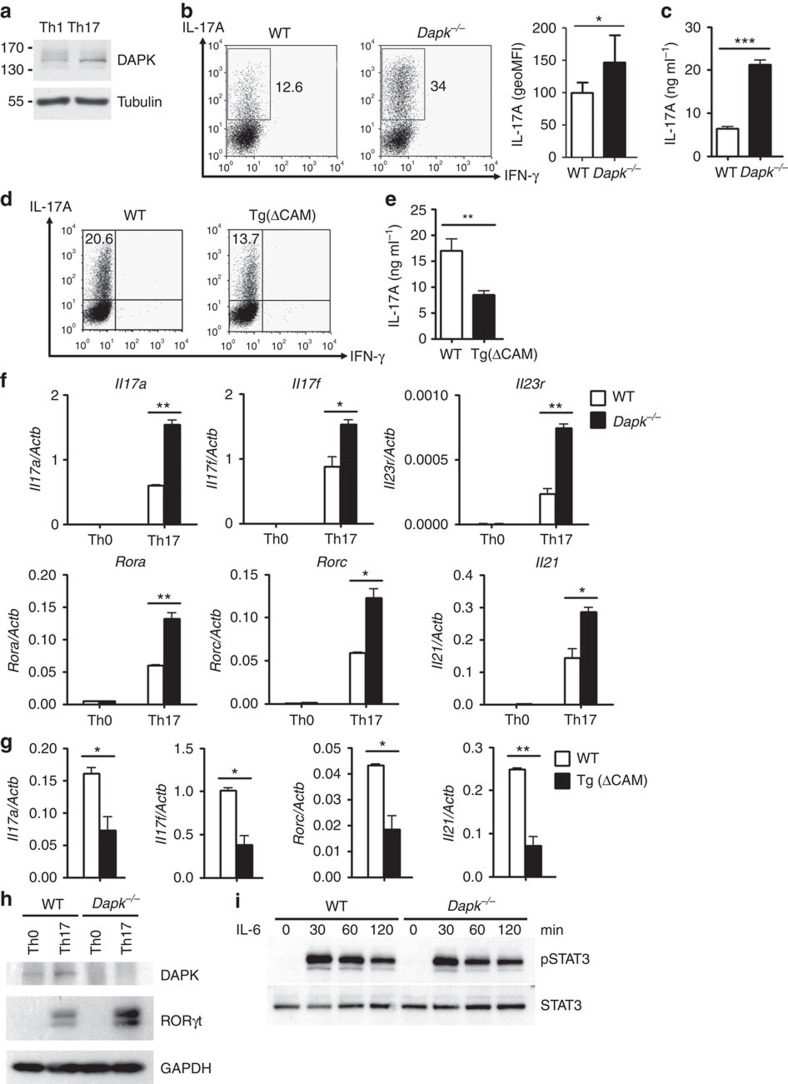
Enhanced Th17 development in *Dapk*^*−/−*^ T cells. (**a**) Increased DAPK levels in Th17 cells. WT naive CD4 (CD4^+^CD25^−^CD44^−^CD62L^+^) T cells were subjected to differentiation into Th1 and Th17 cells for 3 days. The levels of DAPK were determined. (**b**) Increased IL-17 production in *Dapk*^*−/−*^ Th17 cells. WT and *Dapk*^*−/−*^ Th17 cells differentiated for 5 days were re-stimulated with TPA/A23187 for 4 h in the presence of monensin during the last 5 h, and the expression of IL-17 and IFN-γ were analysed by intracellular staining. The numbers represent the percentages of cells positive (left). The geoMFI is calculated (mean±s.d., *n*=7) (right). (**c**) Enhanced IL-17 secretion by *Dapk*^*−/−*^ Th17 cells. WT and *Dapk*^*−/−*^ Th17 cells were re-stimulated with TPA/A23187 for 24 h, and the levels of IL-17 in the supernatant were measured by ELISA (mean±s.d., *n*=3). (**d**,**e**) The [ΔCAM]DAPK transgene inhibited Th17 differentiation. WT and [ΔCAM]DAPK-transgenic T cells were differentiated into Th17 cells for 3 days. The expression of IL-17 and IFN-γ were determined after restimulation by intracellular staining (**d**) or by ELISA (mean±s.d., *n*=3) (**e**). (**f**) Elevated expression of Th17-related genes in *Dapk*^*−/−*^ T cells. WT and *Dapk*^*−/−*^ Th0 and Th17 cells were re-stimulated with TPA/A23187 for 3 h, and RNA was isolated. The expression of *Il17a*, *Il17f*, *Il21*, *Il23r*, *Rorc* and *Rora* were determined by quantitative PCR (mean±s.d., *n*=2). (**g**) Attenuated expression of Th17-associated genes in [ΔCAM]DAPK-transgenic T cells. The expression of *Il17a*, *Il17f*, *Rorc* and *Il21* in WT and [ΔCAM]DAPK-transgenic Th17 cells were determined as in **f**. Mean±s.d., *n*=3. (**h**) Increased expression of RORγt protein in *Dapk*^*−/−*^ Th17 cells. A fraction of the TPA/A23187-re-stimulated cells from **b** were analysed for RORγt expression by western blot. (**i**) Normal STAT3 activation in *Dapk*^*−/−*^ T cells. Freshly isolated WT and *Dapk*^*−/−*^ T cells were stimulated with IL-6 for the indicated times. The levels of phospho-STAT3 and total STAT3 were examined by western blotting. Data (**a**,**c**–**i**) are representative of three independent experiments. *P<0.05, **P<0.01, ***P<0.001 for unpaired *t*-test.

We found that expression of DAPK was higher in Th17 cells than Th1 cells (Fig. 2a), consistent with the report that DAPK is induced by TGF-β^43^. Therefore, we examined differentiation of Th1 and Th17 cells from *Dapk*^*−*/−^ T cells. Naive CD4^+^ T cells from control and *Dapk*^*−*/−^ T cells were stimulated by CD3/CD28 in the presence of TGF-β and IL-6 to induce Th17 differentiation. Differentiation into Th17 was significantly increased for *Dapk*^*−*/−^ T cells, with the IL-17-producing population significantly larger in *Dapk*^*−*/−^ Th17 cells relative to the WT (Fig. 2b, left panel), as also illustrated by an elevated mean fluorescence intensity (MFI; Fig. 2b, right panel). This was also confirmed by ELISA, which showed a threefold increase in IL-17 secretion by *Dapk*^*−*/−^ Th17 cells compared with control Th17 cells (Fig. 2c). In addition, for Th17 cells induced by IL-21 and TGF-β, IL-17 production also increased approximately threefold for *Dapk*^*−*/−^ Th17 cells (Supplementary Fig. 5a). A similar extent of increase in IL-17 expression was found for *Dapk*^*−*/−^ Th17 cells induced by TGF-β, IL-1β, IL-6 and IL-23 (Supplementary Fig. 5b). However, no effect was found on DAPK deficiency in Th17 cells induced by IL-1β, IL-6 and IL-23 (ref. 45; Supplementary Fig. 5c), suggesting that regulation of Th17 by DAPK depends on priming with TGF-β. In parallel experiments, T-cell-specific transgenic expression of [ΔCAM]DAPK attenuated Th17 differentiation mediated by TGF-β and IL-6 (Fig. 2d,e). Together, these results suggest that DAPK inhibits TGF-β-dependent Th17 development.

### Elevated RORγt and RORα in *Dapk* ^*−/−*^ Th17 cells

We then determined whether the increased production of IL-17 correlated with enhanced induction of the transcription of *Il17* genes. Quantitative PCR confirmed that the expression of *Il17a* and *Il17f* was elevated in DAPK-deficient Th17 cells relative to the WT Th17 cells (Fig. 2f), implying that enhanced IL-17 generation could be partly attributed to increased expression of their mRNAs. Induction of *Il21* and *Il23r*—the cytokine and receptor preferentially expressed in Th17 cells, respectively—were also increased in *Dapk*^*−*/−^ Th17 cells. In addition, levels of *Rorc* and *Rora*—the master transcription factors determining the expression of IL-17—were twofold higher in DAPK-null Th17 cells compared with control Th17 cells (Fig. 2f), which correlated with the elevated protein levels of RORγt in *Dapk*^*−*/−^ Th17 cells (Fig. 2h). As a control, in the activated Th0 cells, *Il17a*, *Il17f*, *Il21*, *Il23r*, *Rorc* and *Rora* were absent (Fig. 2f). In addition, the expression of *Il17a*, *Il17f*, *Rorc* and *Il21* was inhibited in [ΔCAM]DAPK-transgenic Th17 cells (Fig. 2g). Together, these results suggest that DAPK suppresses Th17 differentiation by inhibiting the induction of RORγt and RORα.

In parallel experiments, T cells were primed with IL-12 for Th1 differentiation. Supplementary Fig. 5d illustrates that the development into Th1 cells was comparable between WT and *Dapk*^*−*/−^ T cells. Consistent with this result, the expression of *Tbx21* (T-bet) and *Ifng* was not affected by the deficiency of DAPK (Supplementary Fig. 5e). Similarly, differentiation into Th1 cells was not altered by the [ΔCAM]DAPK transgene (Supplementary Fig. 5f).

Since DAPK targets *Rorc* expression, we further examined whether the transcription factors regulating RORγt expression were modulated by DAPK. STAT3 is known to activate the *Rorc* promoter and promote Th17 cell development^12^,^13^,^14^,^15^,^16^,^46^. Figure 2i demonstrates that the levels of STAT3 were comparable between WT and *Dapk*^*−/−*^ T cells. IL-6-triggered STAT3 phosphorylation was also similar between control and *Dapk*^*−/−*^ T cells (Fig. 2i). Similarly, IL-21-triggered STAT3 phosphorylation was comparable between WT and *Dapk*^*−/−*^ T cells (Supplementary Fig. 5g). Therefore, DAPK deficiency does not appear to interfere with STAT3 expression and activation.

We also determined whether activation of the IL-17 promoter by RORγt and RORα was regulated by DAPK. To exclude interference from endogenous DAPK, DAPK was knocked down in 293T cells by siRNA targets to its 5′UTR (Supplementary Fig. 6a). IL-17P-Luc was activated in 293T cells by co-transfection with RORγt (Supplementary Fig. 6b). Co-expression of RORγt with DAPK, the dominant negative DAPK [K42A]DAPK or [ΔCAM]DAPK all inhibited RORγt-directed *Il17* promoter activation. Similarly, RORα-mediated IL-17P-Luc activation was inhibited by co-expression of RORα with DAPK, [K42A]DAPK or [ΔCAM]DAPK (Supplementary Fig. 6c). Therefore, DAPK likely inhibits Th17 development at two sequential stages, that is, expression of both RORγt and RORα as well as RORγt/RORα-mediated IL-17 expression.

### DAPK downregulates HIF-1α in Th17 differentiation

**Figure 3:**
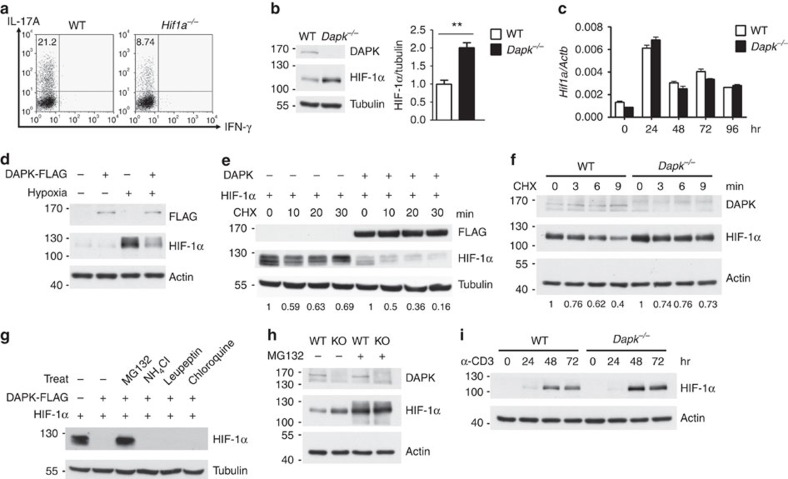
DAPK promotes HIF-1α degradation and DAPK deficiency increases HIF-1α levels in T cells. (**a**) HIF-1α deficiency impairs Th17 differentiation. WT and *Hif1a*^*−/−*^ Th17 cells were re-stimulated and expression of IL-17A and IFN-γ determined. (**b**) Enhanced HIF-1α protein levels in *Dapk*^*−/−*^ Th17 cells. HIF-1α protein contents were determined in Th17 cells differentiated for 3 days (left). HIF-1α levels were quantified and normalized to tubulin (right). Mean±s.e.m., *n*=3. **P<0.01 for unpaired *t*-test. (**c**) DAPK-knockout does not affect HIF-1α mRNA induction during Th17 differentiation. The quantities of *Hif1a* transcript were determined in WT and *Dapk*^*−/−*^ T cells in the course of Th17 differentiation. Mean±s.e.m., *n*=3. (**d**) DAPK induces HIF-1α protein downregulation in normal T cells. Activated primary T cells were transduced with DAPK-FLAG. GFP^+^ T cells were sorted, incubated under hypoxic conditions for 48 h, and the levels of DAPK-FLAG and HIF-lα were determined. (**e**) DAPK reduces HIF-1α protein stability. HEK293T cells were transfected with DAPK and HIF-lα, treated with CHX (50 ng ml^−1^) 48 h later, and the levels of HIF-1α and DAPK were measured. HIF-1α levels were quantified. (**f**) DAPK deficiency increases HIF-1α protein stability in T cells. WT and *Dapk*^*−/−*^ CD4^+^ T cells were differentiated into Th17 cells for 2 days, followed by CHX (25 ng ml^−1^) treatment. The levels of HIF-1α protein were determined by immunoblots and were quantified. (**g**) DAPK-induced HIF-1α degradation is inhibited by MG132. HEK293T cells were transfected with DAPK and HIF-lα. Forty-eight hours later, cells were treated with 10 μM MG132, 200 mM NH_4_Cl, 100 μg ml^−1^ leupeptin, or 100 μM chloroquine for 6 h. The HIF-1α levels were determined. (**h**) DAPK-induced HIF-1α degradation is proteasome-dependent. WT and *Dapk*^*−/−*^ CD4^+^ T cells were differentiated into Th17 cells for 2 days, followed by MG132 (5 μM) treatment for 2 h. The levels of HIF-1α were determined. (**i**) DAPK deficiency increases the induction of HIF-1α protein in T cells. WT and *Dapk*^*−/−*^ T cells were activated with CD3/CD28 under normoxic conditions, and the levels of HIF-1α at the indicated time points were determined. Data are representative of three (**a**,**c**,**e**–**i**) or two (**d**) independent experiments.

It has been previously shown that HIF-1α promotes Th17 differentiation^26^,^27^. Consistent with this observation, diminished Th17 differentiation was found in *Hif1a*^*−/−*^ T cells (Fig. 3a). HIF-1α is known for its participation in carcinogenesis^18^,^19^, while DAPK is a tumour suppressor. Therefore, we investigated whether DAPK directly modulated the expression of HIF-1α. HIF-1α was upregulated in *Dapk*^−/−^ Th17 cells (Fig. 3b), which correlated with the enhanced IL-17 expression in the knockout T cells. The increased HIF-1α protein in *Dapk*^−/−^ T cells was not due to an increase in *Hif1a* mRNA, as *Hif1a* transcript levels were comparable between WT and *Dapk*^−/−^ T cells during the course of Th17 differentiation (Fig. 3c). Because HIF-1α expression is regulated by the mTORC1 pathway^24^,^47^,^48^, we examined whether DAPK deficiency affected mTORC1 signalling in T cells. Expression and phosphorylation of mTOR were similar between WT and *Dapk*^−/−^ Th17 cells (Supplementary Fig. 7a). Further, activation of the mTOR substrates p70 S6 kinase and 4E-BP1 in Th17 cells was not affected by DAPK deficiency (Supplementary Fig. 7a). Similarly, p70 S6 kinase-mediated phosphorylation of ribosomal S6 at S235 and S240 was comparable between WT and *Dapk*^−/−^ T cells (Supplementary Fig. 7b,c). In addition, overexpression of DAPK downregulated the levels of HIF-1α in normal T cells cultured under hypoxic conditions (Fig. 3d). Together, these results suggest that DAPK targets HIF-1α at the post-transcriptional level.

We next examined the effect of DAPK on the stability of HIF-1α. Cycloheximide-induced HIF-1α protein instability was enhanced by the presence of DAPK in 293T cells (Fig. 3e), while HIF-1α protein stability was increased in DAPK-deficient Th17 cells (Fig. 3f), suggesting that DAPK promotes HIF-1α protein destabilization. DAPK-induced HIF-1α protein downregulation was inhibited by MG132 in 293T cells, but not by NH_4_Cl, leupeptin or chloroquine (Fig. 3g). In addition, MG132 treatment increased HIF-1α to similar levels in WT and *Dapk*^−/−^ Th17 cells (Fig. 3h). These results suggest the involvement of the proteasome degradation pathway in DAPK-mediated HIF-1α downregulation in Th17 cells.

In a separate experiment, we examined whether HIF-1α protein expression could be induced by prolonged TCR activation in T cells under normoxic conditions (Fig. 3i). CD3-induced HIF-1α protein expression was much higher in *Dapk*^−/−^ T cells than WT T cells (Fig. 3i), further illustrating the ability of DAPK to downregulate HIF-1α protein.

HIF-1α has also been shown to antagonize Foxp3 and inhibit regulatory T cells (Treg)^27^,^49^. The development and *in vitro* suppressive activity of natural regulatory cells (nTreg) were not affected in *Dapk*^−/−^ nTregs (Supplementary Fig. 8a,b). However, the differentiation of induced regulatory T cells (iTreg) from CD4^+^CD25^−^ T cells was moderately impaired in *Dapk*^−/−^ T cells (Supplementary Fig. 8c). The *in vitro* suppressive activity of *Dapk*^−/−^ iTregs was also reduced relative to WT iTregs (Supplementary Fig. 8d).

### DAPK interacts with cytoplasmic HIF-1α in T cells

**Figure 4:**
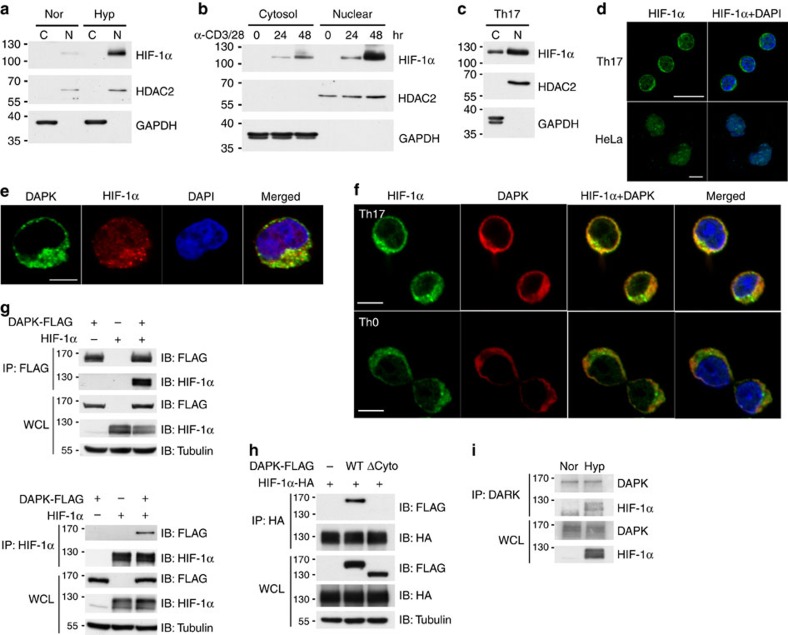
Cytoplasmic presence of HIF-1α and co-localization with DAPK in T cells. (**a**) Predominant nuclear location of HIF-1α in HeLa cells. HeLa cells were cultured in normoxic or hypoxic (1% O_2_) conditions for 24 h. Nuclear (N) and cytoplasmic (C) fractions were isolated, and HIF-1α expression levels were determined. HDAC2 and GAPDH were used as the nuclear marker and cytosolic marker, respectively. (**b**,**c**) Cytoplasmic presence of HIF-1α in T cells. WT T cells were activated with anti-CD3/CD28 for the indicated times (**b**), while naive CD4^+^ T cells were differentiated into Th17 for 48 h (**c**). Nuclear and cytoplasmic fractions were prepared, and HIF-1α expressions were examined. (**d**) Image analysis of HIF-1α localization in Th17 and HeLa cells. Th17 cells (**c**) and HeLa cells (**a**) were stained with anti-HIF-1α and DAPI and analysed by confocal microscopy. Scale bar, 10 μm. (**e**) Co-localization of HIF-1α and DAPK in Jurkat cells. DAPK-FLAG-overexpressing Jurkat cells were cultured under hypoxic (1% O_2_) conditions for 4 h, fixed and stained with anti-FLAG, anti-HIF-1α and DAPI, and analysed by confocal microscopy. Scale bar, 5 μm. (**f**) Co-localization of HIF-1α and DAPK in Th17 and Th0 cells. WT naive T cells were differentiated into Th17 (top) or Th0 cells (bottom) for 2 days, fixed and stained with anti-HIF-1α, anti-DAPK and DAPI, and analysed by confocal microscopy. Scale bar, 5 μm. (**g**) Interaction between HIF-1α and DAPK. HEK293T cells were transfected with DAPK-FLAG or HIF-1α, as indicated. The whole-cell lysates (WCL) were immunoprecipitated with either anti-FLAG (top) or anti-HIF-1α (bottom). The contents of DAPK-FLAG and HIF-1α in the precipitates and lysates were determined. (**h**) Involvement of the DAPK cytoskeleton domain in HIF-1α binding. HEK293T cells were transfected with HIF-1α-HA, and WT DAPK-FLAG or DAPK(ΔCyto)-FLAG. The WCL were immunoprecipitated with anti-HA, and the amounts of DAPK and HIF-1α were determined. (**i**) Association of DAPK with the endogenous HIF-1α in hypoxic T cells. DAPK in normoxic and hypoxic (1% O_2_ for 4 h) Jurkat cells was precipitated by anti-DAPK, and amounts of the associated HIF-1α were determined by western blot. Data are representative of three (**a**–**h**) or two (**i**) independent experiments.

To isolate the mechanism underlying DAPK-dependent HIF-1α degradation, we next examined whether there was a direct interaction between DAPK and HIF-1α. DAPK is a cytosolic protein whereas HIF-1α is known for its predominant nuclear localization, as shown in hypoxic HeLa cells (Fig. 4a). HIF-1α was induced in T cells through prolonged TCR stimulation (Fig. 3i), followed by immunoblotting to determine its localization. Some HIF-1α was present in the cytosolic fraction of activated T cells (Fig. 4b). Similarly, the presence of HIF-1α was detected in the cytoplasmic fraction of Th17 cells (Fig. 4c). The cytosolic localization of HIF-1α in Th17 cells, but not in HeLa cells, was further confirmed by confocal microscopy (Fig. 4d). In addition, DAPK co-localized with HIF-1α in the cytoplasm of hypoxic Jurkat cells (Fig. 4e), as well as in the cytosol of normoxic Th17 and Th0 cells (Fig. 4f).

Since HIF-1α and DAPK co-localized to the cytoplasm, we looked for (and found, see Fig. 4g) their direct interaction by co-immunoprecipitation. The cytoskeleton-binding domain of DAPK was required for the association with HIF-1α (Fig. 4h), while the N-terminal domain of HIF-1α interacted with DAPK (Supplementary Fig. 9a). The association of DAPK with endogenous HIF-1α was also observed in hypoxic T cells (Fig. 4i). Together, these results suggest that DAPK interacts with HIF-1α in the cytosol of T cells, and DAPK may regulate the protein stability of HIF-1α through this direct binding. We also examined whether the kinase domain of DAPK participates in the downregulation of HIF-1α. An N-terminal DAPK fragment containing the kinase domain and ankyrin repeats weakly reduced HIF-1α expression, while a C-terminal DAPK segment from the cytoskeleton binding to death domain effectively promoted HIF-1α degradation (Supplementary Fig. 9b). In addition, we found that HIF-1α is not a kinase substrate of DAPK since recombinant HIF-1α was not phosphorylated by DAPK *in vitro* (Supplementary Fig. 9c,d). Therefore, even though DAPK binds HIF-1α and induces HIF-1α protein instability, the kinase domain of DAPK appears to be disposable in HIF-1α downregulation.

### DAPK induces PHD2-mediated HIF-1α degradation

**Figure 5:**
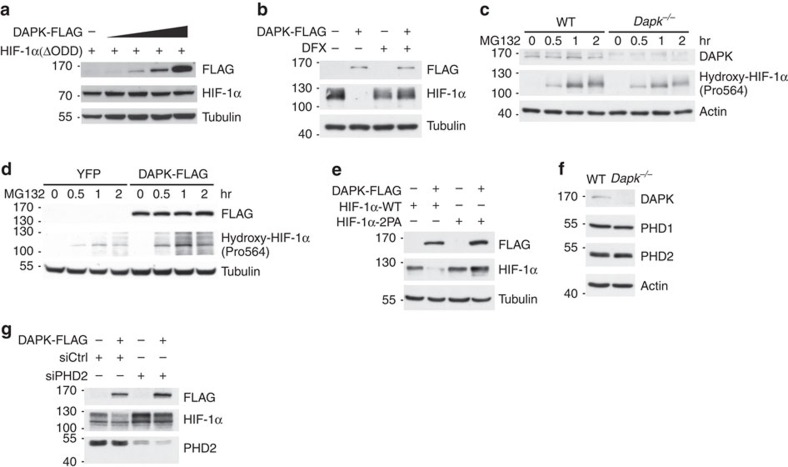
DAPK promotes PHD2-dependent HIF-1α degradation. (**a**) Deletion of the oxygen-dependent degradation (ODD) region confers resistance of HIF-1α to DAPK-induced degradation. HEK293T cells were transfected with DAPK-FLAG and HIF-1α[ΔODD] and the levels of HIF-1α were determined 48 h after transfection. (**b**) PHD inhibitor prevents DAPK-induced HIF-1α degradation in normal T cells. Naive T cells were activated for 48 h and transduced with vector or DAPK-FLAG-IRES-GFP. GFP^+^ T cells were sorted 48 h later, switched to hypoxic incubation (1% O_2_) in the absence or presence of DFX (10 μM) for another 24 h, and the levels of DAPK-FLAG and HIF-1α were determined. (**c**) DAPK deficiency decreases proline hydroxylation of HIF-1α in normal T cells. Naive WT and *Dapk*^*−/−*^ T cells were activated with anti-CD3/CD28 for 48 h in normoxic conditions, followed by treatment with MG132 (10 μM). The extents of Pro564 hydroxylation on HIF-1α were determined at the indicated time points. (**d**) DAPK enhances proline hydroxylation of HIF-1α. YFP control and DAPK-FLAG-expressing Jurkat cells were treated with MG132 (5 μM) in normoxic conditions. The extents of Pro564 hydroxylation on HIF-1α were determined at the indicated time points. (**e**) Mutation of proline hydroxylation sites confers resistance of HIF-1α to DAPK-induced degradation in T cells. Jurkat cells were transfected with DAPK-FLAG, HIF-1α-WT or HIF-1α[P402A/P564A] (HIF-1α-2PA), as indicated. Twenty-four hours after transfection, cells were incubated under hypoxic conditions (1% O_2_) for 6 h, then switched back to normoxia for 10 min, and the levels of HIF-1α were determined. (**f**) DAPK deficiency does not affect the expression of PHD1 and PHD2. WT and *Dapk*^*−/−*^ naive CD4 T cells were allowed to differentiate into Th17 cells for 3 days. Cells were harvested and the expression of PHD1 and PHD2 was examined. (**g**) PHD2-knockdown prevents DAPK-induced HIF-1α degradation in T cells. Jurkat T cells were transfected with DAPK-FLAG, siRNA control (siCtrl), or siRNA for PHD2 (siPHD2). Twenty-four hours later, cells were switched to hypoxic conditions for 6 h, and the levels of HIF-1α, PHD2 and HSP90 were determined. Data are representative of three (**a**,**d**) or two (**b**,**c**,**e**–**g**) independent experiments.

The inclusion of ubiquitin enhanced the ability of DAPK to downregulate HIF-1α (Supplementary Fig. 10a), illustrating the involvement of the ubiquitin-dependent degradation process. The stability of HIF-1α protein is regulated by proline hydroxylation, a step necessary for its subsequent binding to VHL protein leading to ubiquitination and proteasome degradation^18^,^19^,^20^,^22^. Therefore, we sought to identify the degradation pathways responsible for DAPK-regulated HIF-1α stability. Deletion of the HIF-1α oxygen-dependent degradation domain containing the proline residues necessary for hydroxylation conferred resistance to DAPK-induced degradation (Fig. 5a). Similarly, inhibition of PHD1/2, which hydroxylates HIF-1α protein, by deferoxamine mesylate (DFX) restored the expression levels of HIF-1α protein in DAPK-expressing T cells and in 293T cells (Fig. 5b; Supplementary Fig. 10b). This result was consistent with the decreased hydroxylation of HIF-1α protein at proline 564 in *Dapk*^*−/−*^ T cells (Fig. 5c) and the increased proline 564 hydroxylation in DAPK-overexpressing Jurkat cells (Fig. 5d). Mutation of the hydroxylation sites Pro402 and Pro564 on HIF-1α into alanine (2PA) conferred resistance of HIF-1α protein to DAPK-induced degradation in T cells and in HEK293T cells (Fig. 5e; Supplementary Fig. 10c). We confirmed that DAPK deficiency did not affect the levels of PHD1 and PHD2 in T cells (Fig. 5f). However, knockdown of PHD2 prevented DAPK-induced HIF-1α degradation in T cells (Fig. 5g). Taken together, these results suggest that DAPK-induced HIF-1α downregulation is partly mediated by PHD2-triggered proline hydroxylation on HIF-1α.

**Figure 6:**
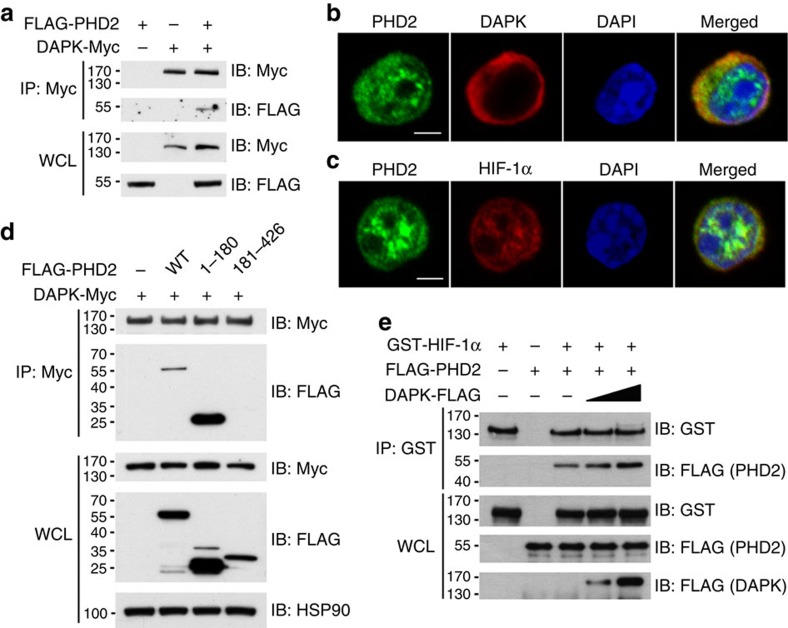
DAPK interacts with PHD2 and increases PHD2-HIF-1α association. (**a**) Interaction of DAPK with PHD2. Jurkat cells were transfected with FLAG-PHD2 and DAPK-Myc. The whole-cell lysates (WCL) were immunoprecipitated with anti-Myc, and the contents of FLAG-PHD2 and DAPK-Myc in the precipitates and WCL were determined by anti-FLAG and anti-Myc. (**b**) Co-localization of DAPK with PHD2 in Th17 cells. WT naive T cells were differentiated into Th17 for 2 days, and cells were fixed. Fixed cells were stained with anti-PHD2, anti-DAPK and DAPI, and analysed by confocal microscopy. Scale bar, 2.5 μm. (**c**) Co-localization of HIF-1α with PHD2 in Th17 cells. Th17 cells were stained with anti-PHD2, anti-HIF-1α and DAPI, and analysed by confocal microscopy. Scale bar, 2.5 μm. (**d**) DAPK interacts with the N-terminal domain of PHD2. HEK293T cells were transfected with DAPK-Myc, and the N-terminal (aa 1–180) or C-terminal (aa 181–426) domain of FLAG-PHD2 as indicated. Cell lysates were immunoprecipitated with anti-Myc, and the contents of PHD2 and DAPK were determined. (**e**) DAPK increases the association of PHD2 with HIF-1α *in vitro*. Recombinant GST-HIF-1α protein was incubated with FLAG-PHD2 in the absence or presence of recombinant DAPK-FLAG proteins in PBS at 4 °C for 4 h. The GST-HIF-1α complex was pulled down by anti-GST, and the contents of FLAG-PHD2, GST-HIF-1α and DAPK-FLAG in the precipitates and incubation mixtures were determined. Data (**a**–**e**) are representative of two independent experiments.

We further detected an interaction between DAPK and PHD2 in T cells (Fig. 6a). Co-localization of DAPK with PHD2 was observed in Th17 cells by confocal microscopy (Fig. 6b). In addition, PHD2 co-localized with HIF-1α in the nucleus and cytoplasm of Th17 cells (Fig. 6c). We also identified the N-terminal part (aa 1–180) of PHD2 as the DAPK-interacting region (Fig. 6d). Addition of recombinant DAPK protein enhanced the interaction of GST-HIF-1α and FLAG-PHD2 in an *in vitro* binding assay (Fig. 6e). Therefore, by binding to both HIF-1α and PHD2, a possible role for DAPK is to enhance the association of PHD2 with HIF-1α and increase HIF-1α degradation.

### HIF-1α knockout inhibits *Dapk* ^*−/−*^ Th17 cell differentiation

**Figure 7:**
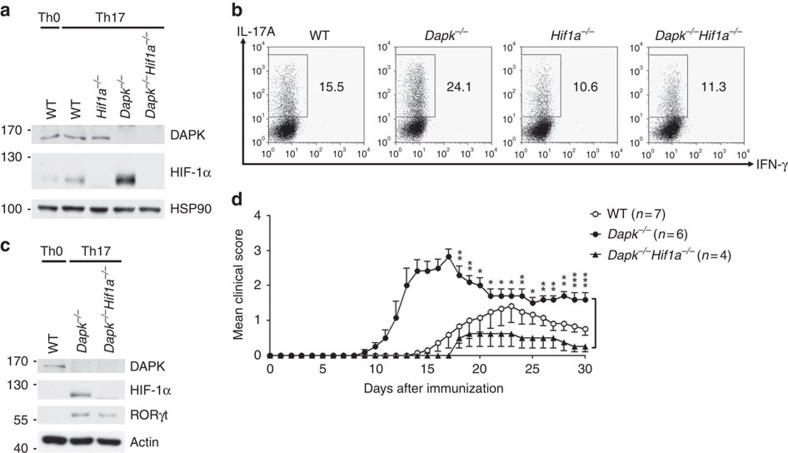
HIF-1α-knockout prevents excess Th17 differentiation in *Dapk*^*−/−*^ T cells. Naive CD4^+^ T cells from WT, *Dapk*^*−/−*^, *Hif1a*^*−/−*^ and *Dapk*^*−/−*^*Hif1a*^*−/−*^ mice were allowed to differentiate into Th0 and Th17 cells. (**a**) The levels of DAPK and HIF-1α were determined in Th17 cells and Th0 cells after 3 days of differentiation. (**b**,**c**) HIF-1α deficiency reduces the excess Th17 differentiation in *Dapk*^*−/−*^ T cells. TPA/A23187-stimulated production of IL-17A and IFN-γ was examined in Th17 cells after 5 days of differentiation (**b**), and the contents of HIF-1α and RORγt were determined (**c**). Data are representative of three (**b**) or two (**a**,**c**) independent experiments. (**d**) HIF-1α deficiency prevents exacerbated EAE generation in *Dapk*^*−/−*^ mice. EAE was induced in WT, *Dapk*^*−/−*^, *Hif1a*^*−/−*^ and *Dapk*^*−/−*^*Hif1a*^*−/−*^ mice as in Fig. 1a. Mice were monitored for clinical signs of paralysis. Values are mean±s.e.m., *P<0.05, **P<0.01, ***P<0.001 for unpaired *t*-test.

We hypothesized that if the excess generation of IL-17 in *Dapk*^*−/−*^ T cells is due to enhanced HIF-1α-mediated Th17 differentiation, the additional deletion of HIF-1α should reduce IL-17 production to normal levels in *Dapk*^*−/−*^ T cells. We generated mice with *Dapk*^*−/−*^ *Hif1a*^*−/−*^ by crossing mice with T-cell-specific deletion of HIF-1α (*Cd4*^*Cre*^ X *Hif1a*^*f/f*^) with *Dapk*^*−/−*^ mice. Deletion of HIF-1α and/or DAPK in T cells from each genotype was confirmed by immunoblotting (Fig. 7a). In agreement with our hypothesis, we found that while *Dapk*^*−/−*^ T cells exhibited enhanced differentiation of naive CD4^+^ T cells into Th17 cells, *Dapk*^*−/−*^ *Hif1a*^*−/−*^ T cells displayed levels similar to WT T cells (Fig. 7b). Knockout of HIF-1α also decreased the levels of RORγt in *Dapk*^*−/−*^ Th17 cells (Fig. 7c). Therefore, elevated HIF-1α levels partly account for the increased IL-17 expression in *Dapk*^*−/−*^ T cells. We further examined whether selective deletion of HIF-1α in T cells attenuated the exacerbated EAE generation in *Dapk*^*−/−*^ mice. The severe EAE disease scores in *Dapk*^*−/−*^ mice were largely attenuated by T-cell-selective deletion of HIF-1α (Fig. 7d), supporting that DAPK acts upstream of HIF-1α in EAE development. T-cell-specific deletion of HIF-1α did not affect the development and suppressive activity of nTreg in *Dapk*^*−/−*^ mice (Supplementary Fig. 11a,b). The reduced development of *Dapk*^−/−^ iTregs was restored by HIF-1α knockout (Supplementary Fig. 11c), yet the impaired suppressive activity was not corrected in *Dapk*^*−/−*^*Hif1a*^*−/−*^ iTregs (Supplementary Fig. 11d). Together, these results suggest that DAPK–HIF-1α interaction contributes little to maintain normal Treg functioning, in contrast to a prominent role of DAPK–HIF-1α antagonism in Th17 cells.

## Discussion

In this study, we identified a negative role of DAPK in Th17 development. In *Dapk*^*−*/−^ T cells, generation of IL-17A, IL-17F, IL-21 and IL-23R were highly elevated. We further found that the levels of RORγt and RORα—the transcription factors dictating IL-17 expression—were elevated in DAPK-deficient Th17 cells; even though activation of the *Rorc* inducer STAT3 was similar between WT and *Dapk*^*−*/−^ T cells (Fig. 2i; Supplementary Fig. 5g). Instead, increases in the expression of RORγt and RORα could be partly attributed to elevated levels of HIF-1α protein. In addition, phosphorylation of mTOR, p70 S6 kinase, 4E-BP1 and ribosomal S6 were comparable between WT and *Dapk*^−/−^ Th17 cells (Supplementary Fig. 7), suggesting that the increased levels of HIF-1α in *Dapk*^−/−^ Th17 cells are not caused by increased mTOR signalling. We further found that DAPK promoted the destabilization of HIF-1α protein in a proteasome-dependent manner (Fig. 3).

DAPK expression is induced by TGF-β (ref. 43), which is commonly used for Th17 differentiation^10^,^11^. This may partly explain why DAPK levels were higher in Th17 cells than Th1 cells (Fig. 2a). The lower DAPK expression in Th1 cells likely correlated with the weak effect of DAPK deficiency in Th1 cells. Even though naive *Dapk*^*−/−*^ T cells displayed increased production of IFN-γ (Supplementary Fig. 2b), IFN-γ expression was not enhanced in re-stimulated DAPK-deficient T cells, including T cells optimized for Th0 and Th1 differentiation (Supplementary Figs 2d and 5d). In addition, IFN-γ production was comparable in MOG-primed T cells from WT and *Dapk*^*−*/−^ mice (Fig. 1e). Similarly, transgenic expression of DAPK did not affect Th1 development (Supplementary Fig. 5f).

DAPK deficiency led to enhanced sensitivity to EAE induction (Fig. 1a–c). We found that the ratio of IL-17-expressing cells to IFN-γ-expressing cells was significantly increased in spinal cords from *Dapk*^*−*/−^ mice during peak EAE induction (Supplementary Fig. 4), suggesting enhanced spinal cord infiltration of *Dapk*^*−*/−^ Th17 cells. We also observed that transfer of *Dapk*^*−*/−^ 2D2 Th17 cells induced highly exacerbated EAE in comparison to WT Th17 cells (Fig. 1f). DAPK-deficient Th17 cells thus displayed increased encephalitogenic activity relative to WT Th17 cells. In contrast, EAE induction by *Dapk*^*−*/−^ 2D2 Th1 cells was similar to that induced by WT 2D2 Th1 cells (Fig. 1g). Therefore, DAPK deficiency correlates with preferential induction of pathogenic Th17.

We have previously shown that DAPK specifically suppresses TCR-induced NF-κB activation^42^, as confirmed here by the elevated RelA nuclear translocation in *Dapk*^*−/−*^ T cells (Supplementary Fig. 2e). NF-κB activation in T cells is required for the generation of autoimmune encephalomyelitis; mice deficient in NF-κB1 (p50) are resistant to the induction of EAE^50^, whereas PKCθ deficiency prevents T-cell activation and the pathogenesis of EAE^51^. NF-κB has been shown to directly activate the *Hif1a* promoter^52^. In the present study, we found that induction of *Hif1a* transcripts during Th17 differentiation was not affected by *Dapk* deficiency (Fig. 3c), indicating that enhanced NF-κB activation in *Dapk*^*−/−*^ T cells did not lead to a further increase in *Hif1a* expression. We speculate that the levels of NF-κB activation in WT T cells were already sufficient for optimal transcriptional activation of *Hif1a* mRNA.

A previous study of DRAK2 showed that deletion of the gene *Stk17b2,* which encodes DRAK2, results in complete resistance to EAE induction^35^. DRAK2 knockout is accompanied by increased T-cell activation, similar to *Dapk*-deficient T cells^35^. However, TCR-induced NF-κB activation is selectively enhanced in *Dapk*^−/−^ T cells (Supplementary Fig. 2e), but is attenuated in DRAK2-null T cells^35^. In addition, DRAK2 consists only of a catalytic domain, lacking the cytoskeleton-binding domain^33^ by which DAPK interacts with HIF-1α (Fig. 4h). We also found that a DAPK kinase domain fragment was not efficient in promoting HIF-1α degradation (Supplementary Fig. 9b). It is likely that the different roles in EAE induction of DAPK and DRAK2 could be partly attributed to their differential effects on NF-κB activation and HIF-1α downregulation.

A novel finding in the present study is that DAPK interacts with HIF-1α and promotes the degradation of HIF-1α in T cells. We reveal that, in contrast to the predominant nuclear localization of HIF-1α in HeLa cells, HIF-1α protein was present in both the cytoplasm and nucleus of T cells (Fig. 4a–f). The cytosolic location of HIF-1α protein permits direct interaction with, and subsequent destabilization by, DAPK. Therefore, DAPK exhibits an additional ability to regulate HIF-1α protein in T cells by its ability to associate with HIF-1α. We further show that DAPK increased the proline hydroxylation of HIF-1α protein (Fig. 5c,d), and HIF-1α proteins lacking the hydroxylation sites were resistant to DAPK-induced degradation (Fig. 5a,e). Knockdown of PHD2 also eliminated the ability of DAPK to downregulate HIF-1α (Fig. 5g), suggesting that the binding of DAPK to HIF-1α protein leads to proline hydroxylation of HIF-1α and subsequent HIF-1α ubiquitination by VHL.

HIF-1α is pivotal for Th17 differentiation. The specific downregulation of HIF-1α protein by DAPK leads to preferential enhancement of Th17 development in the absence of DAPK-null T cells. This is further supported by the fact that additional HIF-1α knockout eliminated the excess Th17 differentiation and encephalitogenic activity of *Dapk*^*−*/−^ T cells (Fig. 7). HIF-1α protein is also known to promote Foxp3 degradation and negatively regulates Treg differentiation^27^,^49^. Despite that differentiation and functioning of iTreg from *Dapk*^*−*/−^ T cells were impaired (Supplementary Fig. 8c,d), knockout of HIF-1α did not restore functioning of *Dapk*^*−*/−^ iTregs (Supplementary Fig. 11d), suggesting a minor role of DAPK–HIF-1α antagonism in Treg cells. Therefore, the ability of DAPK to inhibit HIF-1α predominantly regulates Th17 cells, and impaired Treg function do not seem to contribute to the enhanced Th17 development and severe EAE disease observed in *Dapk*^*−*/−^ mice. Why Th17 and Tregs display different susceptibility to regulation by the DAPK–HIF-1α interaction is currently being investigated.

Recent studies have revealed that hypoxia promotes the expression of miR-103/107 that target DAPK for downregulation in cancer cells^53^. HIF-1α induces the expression of KLHL20, which coordinates with Cul3 for ubiquitination and proteasome degradation of DAPK in several cancer cell lines^54^,^55^. Together with the results from the present study, we propose a mutual antagonism between HIF-1α and DAPK. We suggest that the continuous cross-interaction between HIF-1α and DAPK, modulated by specific environmental cues, may determine the patho-physiological status of Th17 cells.

DAPK is a tumour suppressor known to be downregulated in many types of cancers^36^,^37^,^38^,^39^,^40^,^41^. Our results illustrate that DAPK is not only a tumour suppressor, but also plays a role in the prevention of Th17-mediated autoimmune diseases like EAE. DAPK suppresses tumour formation and metastasis through its promotion of apoptosis, induction of autophagy and regulation of cell motility^56^. In the present study, we have demonstrated that DAPK inhibits EAE development by inducing HIF-1α degradation and inhibiting pro-inflammatory Th17 cell differentiation. Notably, HIF-1α is also known for its prominent role in tumorigenesis, with levels of HIF-1α correlating with poor prognoses for various cancer types^18^,^19^. Even though HIF-1α is exclusively localized in the nucleus of most cancer cells, it will be interesting to examine whether the ability of DAPK to promote HIF-1α degradation in the cytoplasm also contributes to the tumour suppressing function of DAPK in selective cancer types. We found that HIF-1α was present in the cytoplasm of T-cell leukaemia Jurkat cells and was subjected to DAPK regulation (Figs 4e and 5e), suggesting that DAPK likely exhibits a tumour suppressor effect in T leukaemia cells by directly binding to the cytosolic HIF-1α to prompt HIF-1α downregulation.

Th17 cells contribute to autoimmune diseases—including multiple sclerosis, rheumatoid arthritis, inflammatory bowel diseases, psoriasis and autoimmune diabetes^1^,^2^,^3^, and Th17 is a target for immunotherapy in these inflammatory diseases. In addition to promoting Th17 differentiation, HIF-1α contributes to the inflammatory activities of different innate immune cells including macrophages and neutrophils^22^. Therefore, targeted HIF-1α degradation by DAPK upregulation is a potential novel approach in the treatment of Th17-associated inflammatory diseases.

The critical role of HIF-1α in carcinogenesis and metastases has led to the identification of drugs that inhibit HIF-1α (refs 18, 19, 21, 31, 32). Conceivably, HIF-1α inhibitors are also potential therapeutic agents for HIF-1α-mediated diseases—including pulmonary arterial hypertension, hereditary erythrocytosis, obstructive sleep apnea, ocular neovascularization and traumatic shock^19^,^20^—as well as Th17-mediated inflammatory diseases. However, HIF-1α is critical for oxygen homoeostasis, and is protective in coronary artery disease, peripheral artery disease, wound healing, colitis and organ transplantation rejection^19^,^20^,^21^. Thus, the therapeutic benefits of HIF-1α inhibitors are expected to be offset by the loss of HIF-1α-mediated protective responses. In the present study, we used Th17 as an example to demonstrate an alternative approach to downregulating HIF-1α. The cytoplasmic presence of HIF-1α permits its interaction with DAPK in the cytosol of T cells, leading to HIF-1α downregulation. DAPK-induced cytosolic HIF-1α degradation presumably does not operate in cells exhibiting exclusively nuclear HIF-1α, leaving the HIF-1α in those cells unaffected. Compounds that activate DAPK have been identified^41^, and further improvements in the specificity of drugs for DAPK induction may lead to reagents capable of effectively downregulating HIF-1α in DAPK-dependent processes. These drugs could potentially be used for treatment of Th17-mediated inflammatory diseases and T-cell leukaemia. Future investigations will help validate the therapeutic approach of inhibiting HIF-1α pathogenic activity in T cells through DAPK upregulation, without affecting the HIF-1α in other cells.

## Methods

### Reagents

Calcium ionophore (A23187), phorbol 12-myristate 13-acetate (TPA), MG132, chloroquine, ammonium chloride (NH_4_Cl), leupeptin, cycloheximide (CHX), DFX, anti-DAPK (D2178, DAPK-55, western blot (WB) 1:1,000, immunofluorescence (IF) 1:50), anti-HA (H3633, HA-7, WB 1:1,000, immunoprecipitation (IP) 1 μg per test), and anti-FLAG (F1804, M2, WB 1:1,000, IF 1:400) were purchased from Sigma (St Louis, MO, USA). Anti-p70S6K (sc-230, C-18, WB 1:1,000), anti-GAPDH (sc-32233, 6C5, WB 1:10,000), anti-HDAC2 (sc-55541, B-2, WB 1:500) and anti-p65 (sc-372G, C-20, WB 1:1,000) were obtained from Santa Cruz Biotech (Santa Cruz, CA, USA). Anti-RORγt (14–6981, B2D, WB 1:1,000), anti-CD4-PE-Cy7 (25-0041, GK1.5, flow cytometry (FCM) 1:100), anti-CD62L-APC (17–0621, MEL-14, FCM 1:100), recombinant mouse IL-6 (14–8061) and IL-23 (14–8231), anti-CD69-PE (12–0691, H1.2F3, FCM 1:100), anti-Foxp3-APC (50–5773, FJK-16s, FCM 1:100), mouse IL-2 and mouse IFN-γ ELISA kit were purchased from eBioscience (San Diego, CA, USA). STAT3 antibody (GTX15523, WB 1:1,000) was obtained from GeneTex (Irving, CA, USA). Anti-phospho STAT3^Y705^ (9131, WB 1:1,000), anti-phospho mTOR^S2448^ (2971, WB 1:1,000), anti-phospho p70S6K^T389^ (9205, WB 1:1,000), anti-phospho 4E-BP1^T70^ (9455, WB 1:1,000), Alexa 647-conjugated anti-S6^S235/236^ (4851, D57.2.2E, FCM 1:100), Alexa 647-conjugated anti-S6^S240/244^ (5044, D68F8, FCM 1:100), anti-mTOR (2972, WB 1:1,000), anti-4E-BP1 (9452, WB 1:1,000), anti-PHD2 (4835, D31E11, WB 1:1,000, IF 1:50), anti-Myc (2276, 9B11, WB 1:1,000, IP 1 μg per test), and anti-hydroxy HIF-1α^P564^ (3434, D43B5, WB 1:1,000) were purchased from Cell Signaling Technology (Danvers, MA, USA). Anti-actin (MAB1501, C4, WB 1:6,000) and anti-tubulin (05–661, AA2, WB 1:6,000) were purchased from Merck Millipore (Billerica, MA, USA). Anti- mouse HIF-1α (10006421, WB 1:1,000, IF 1:100) was purchased from Cayman Chemicals (Ann Arbor, MI, USA). Anti-human HIF-1α (610959, 54/HIF-1α, WB 1:500, IF 1:100), and anti-HSP90 (610419, 68/Hsp90, WB 1:4,000) were obtained from BD-Biosciences (Franklin Lakes, NJ, USA). Anti-CD4-FITC (100501, RM4–5, FCM 1:100), anti-CD8a-PE (100708, 53-6.7, FCM 1:100), anti-CD44-FITC (103006, IM7, FCM 1:100), anti-IL-17A-PE (506904, TC11-18H10.1, FCM 1:100), anti-TCRβ-FITC (109206, H57–597, FCM 1:100), anti-CD25-PE (102008, PC61, FCM 1:100), anti-IFN-γ-APC (505810, XMG1.2, FCM 1:100), anti-IFN-γ (507801, DB-1, IF 1:200), LEAF purified anti-mouse IFN-γ (505707, R4-6A2, cell culture 5 μg ml^−1^) and LEAF purified anti-mouse IL-4 (504115, 11B11, cell culture 5 μg ml^−1^) were purchased from BioLegend (San Diego, CA, USA). Anti-PHD1 (ab108980, EPR2745, WB 1:1,000) and anti-IL-17 (ab91649, IF 1:100) were purchased from Abcam (Cambridge, UK). Anti-mouse CD3 (2C11), anti-mouse CD28 (37.51), anti-mouse IL-2, and anti-mouse CD4 (RL172.4) were purified in our laboratory. Recombinant mouse IL-12 and IL-21, mouse IL-17A ELISA kit, and mouse IL-4 ELISA kit were purchased from R&D (Minneapolis, MN, USA). Recombinant mouse IL-2 and human TGF-β were purchased from PeproTech (Rocky Hill, NJ, USA). Anti-HIF-1α (A300-286A, IP 1 μg per test) was purchased from Bethyl Laboratories (Montgomery, TX, USA). Mouse anti-Lamin B1 (33–2000, L-5, WB 1:1,000), Alexa Flour488-labelled goat anti-mouse IgG (A11001, IF 1:500), Alexa Flour555-labelled goat anti-mouse IgG (A21422, IF 1:500), Alexa Flour488-labelled goat anti-rabbit IgG (A11034, IF 1:500) and Alexa Flour555-labelled goat anti-rabbit IgG (A21428, IF 1:500) were purchased from Thermo Fisher Scientific (Waltham, MA, USA). siRNA control (siCtrl), siRNAs specific for PHD2 (EGLN1 siRNA SMARTpool) and for human DAPK (siDAPK) were purchased from GE Dharmacon (Lafayette, CO, USA). The sequence of siDAPK is: UCU GGG AAG CGG AGC UGA AUU.

### Cell cultures

HEK293T (ATCC CRL-3216), HeLa (ATCC CCL-2) and Jurkat T lymphoma (clone E6-1, ATCC TIB-152) were obtained from ATCC. Cell lines were examined for mycoplasma contamination using Mycoplasma Detection Kit (R&D). Normal T cells and Jurkat cells were cultured in RPMI-1640 medium supplemented with 10% fetal calf serum (Invitrogen Life Technology), 10 mM glutamine, 100 U ml^−1^ penicillin, 100 μg ml^−1^ streptomycin, and 50 μM 2-ME (complete RPMI medium). HEK293T and HeLa cells were cultured in DMEM medium with the same supplements as in complete RPMI medium. For hypoxia (1% O_2_) experiments, T cells were cultured in the cell incubator containing an oxygen controller ProOx-110 (BioSpherix) using gas of 5% CO_2_ and 95% N_2_ to reduce the oxygen content. Expression of DAPK or its mutants was performed by transfection of T cells with DAPK, [K42A]DAPK, or [ΔCAM]DAPK through electroporation using the MP-100 system (Life Technologies).

### Mice

Production of *Dapk*^−/−^ mice^44^ and p37.1-[ΔCAM]DAPK-transgenic mice^42^ (both in a C57BL/6 background) were previously described. B6.129-*Hif1a*^*tm3Rsjo*^/J (*Hif1a*^*f/f*^) mice^57^, *Rag1*^*−/−*^ mice (in C57BL/6 background), and C57BL/6-Tg(Tcra2D2, Tcrb2D2)1Kuch/J (2D2 TCR^MOG^) transgenic mice^58^ were obtained from Jackson Laboratories (Bar Harbor, ME, USA). *Cd4-Cre* mice (in C57BL/6 background) were purchased from Taconic Farms (Hudson, NY, USA). Mice were maintained in the SPF mouse facility of the Institute of Molecular Biology, Academia Sinica. All mouse experiments were performed using 8–16-week-old mice. For *in vivo* experiments, sex-matched male and female mice were used. Donor and recipient mice were also sex-matched in T cells transfer assay. Because mouse experiments are limited by the availability of the knockout mice and their littermate control, no randomization was used. Mouse experiments were not blinded. All mouse experiments were conducted with approval from the Institutional Animal Care & Utilization Committee, Academia Sinica.

### Induction of T-helper cells and iTregs

Mouse T cells were isolated from spleen and peripheral lymph nodes by negative selection using an anti-mouse Ig panning method. Total CD4^+^ T cells were purified by positive selection using a rat anti-mouse CD4 antibody (RL172.4) panning method. Naive CD4 T cells (CD4^+^CD25^−^CD44^low^CD62L^high^) were then sorted by FACSVantage SE (BD Bioscience). Naive CD4 T cells were then activated by 2 μg ml^−1^ plate-bound anti-CD3 (2C11) plus 1 μg ml^−1^ anti-CD28 (37.51). IL-2 (20 U ml^−1^) was added for Th0 differentiation; IL-12 (10 ng ml^−1^), IL-2 (5 ng ml^−1^) and anti-IL-4 (10 μg ml^−1^) were added to induce Th1 development; and TGF-β (2 ng ml^−1^), IL-6 (2 ng ml^−1^), anti-IL-2 (5 μg ml^−1^), anti-IL-4 (5 μg ml^−1^) and anti-IFN-γ (5 μg ml^−1^) were added to induce Th17 differentiation. Th17 cells were also induced by replacing IL-6 with IL-21 (100 ng ml^−1^) in the same mixture. After 3-to-5 days, Th1 and Th17 cells were re-stimulated with anti-CD3/CD28 or TPA/A23187. For intracellular staining, monensin (2 nM) was added 1 h after activation, and intracellular content of IL-17 and IFN-γ was determined after another 3 h. For mRNA expression, cells were harvested 3 h after TPA/A23187 stimulation and RNA was prepared. For ELISA, cell culture supernatants were collected 24 h after TPA/A23187 activation. Induced Treg (iTreg) cells were generated from CD4^+^CD25^−^ T cells and activated by IL-2 (20 ng ml^−1^), TGF-β (5 ng ml^−1^), and plate-bound anti-CD3 (2 μg ml^−1^) plus anti-CD28 (1 μg ml^−1^) for 1-to-5 days.

### EAE induction

DAPK[ΔCAM]-transgenic, DAPK-knockout, and their WT littermate control mice were immunized subcutaneously with 200 μg of MOG peptide 35–55 (Kelowna, Taipei, Taiwan) emulsified in CFA containing 400 μg *Mycobacterium tuberculosis* (Sigma), followed by intraperitoneal injection of 200 ng pertussis toxin (List Labs, Campbell, CA, USA) at day 0 and day 2. Mice were monitored daily for clinical signs and then clinically scored by the following criteria: 0, no syndrome; 1, limp tail; 1.5, tail paralysis and waddling gait; 2, weak tail and partial hind limb paralysis; 2.5, paralysis of one hind limb; 3, total hind limb paralysis; 3.5, complete hind limb and partial fore limb paralysis; 4, moribund state; 5, complete paralysis and death. Numbers of mice used in EAE experiments were based on those used in documented EAE studies^27^,^59^,^60^,^61^.

EAE induced by adoptive transfer of 2D2 T cells was performed according to the protocol of Jäger *et al.*^59^. Naive T cells from 2D2 or 2D2 *Dapk*^*−/−*^ mice were differentiated into Th17 cells by incubation with MOG_35-55_ peptide (20 μg ml^−1^), hTGF-β (3 ng ml^−1^), mIL-6 (20 ng ml^−1^), anti-IL-4 (5 μg ml^−1^), anti-IFN-γ (5 μg ml^−1^) and threefold γ-irradiated T-cell-depleted splenic cells. IL-23 (10 ng ml^−1^) was added 48 h after differentiation. Five days after initial Th17 differentiation, Th17 cells were re-stimulated with plate-bound anti-CD3/anti-CD28 (2 μg ml^−1^each) in the absence of exogenous cytokines for 48 h. 2D2 or 2D2 *Dapk*^*−/−*^ naïve T cells were differentiated into Th1 by incubation with MOG_35-55_ peptide (20 μg ml^−1^), mIL-12 (10 ng ml^−1^), anti-IL-4 (10 μg ml^−1^) and threefold γ-irradiated T-cell-depleted splenic cells for 5 days. Th17 and Th1 cells were washed three times with cold PBS, and 5 × 10^6^ Th17 cells or 3 × 10^6^ Th1 cells were transferred intravenously into each *Rag1*^*−/−*^ mouse. Recipient mice were injected i.p. with 200 ng of pertussis toxin at day 0 and day 2 after T cells transfer. The classical EAE scores were described as above. The non-classical (atypical) EAE was scored by the following criteria^60^: 0, no syndrome; 1, head turned slightly (ataxia, no tail paralysis); 2, head-turn more pronounced; 3, inability to walk in a straight line; 4, laying on side and rolling continuously unless supported; 5, death. Half scores were given (i.e., 1.5, 2.5) when the clinical phenotype was deemed to lie between two defined criteria.

### Histology on spinal cord sections

Mice were perfused with phosphate buffer saline (PBS) followed by 4% (v/v) paraformaldehyde (PFA) in PBS. Spinal cords were isolated and fixed with 4% (v/v) PFA in PBS overnight at 4 °C. Spinal cords were dehydrated with 30% sucrose in PBS for 48 h at 4 °C. Spinal cords were embedded in optimal cutting temperature compound and serial cryosections (30 μM thick) were acquired. Tissue sections were stained with hematoxylin & eosin or luxol fast blue. Images were obtained on a Zeiss Axio Imager Z1 microscope (Jena, Germany) with an objective lens of plan-Apochromat 10 × /0.45 M27 and the camera of Axiocam 506. The channel was TL Brightfield and the depth of focus was at 5.43 μM.

For staining with anti-IFNγ and anti-IL-17, PFA-fixed optimal cutting temperature-embedded sections were pretreated with sodium citrate buffer (pH 6.0, 10 mM sodium citrate, 0.05% Tween-20) at 55 °C for 30 min for antigen retrieval. Sections were washed and permeabilized with 0.3% (v/v) Triton X-100 in PBS at room temperature for 30 min. Sections were incubated in blocking buffer (1% BSA, 0.3% Triton X-100 in PBS) at room temperature for 30 min. Sections were then incubated with primary antibodies diluted in blocking buffer for 48 h at 4 °C. The primary antibodies were anti-IL-17 (Abcam, ab91649, 1:100 dilution) and anti-IFNγ (BioLegend, DB-1, 1:200 dilution). Sections were washed and incubated with Alexa Flour 488 or 555 secondary antibodies (Invitrogen, 1:500 dilution) at room temperature for 2 h. Samples were then washed and mounted with DAPI Fluoromount-G buffer (SouthernBiotech, Birmingham, AL, USA).

### Quantitative PCR

T cells were harvested at the time indicated and total RNA was isolated using Trizol (Invitrogen). cDNAs were prepared and analysed for the expression of the gene of interest by real-time PCR using a SYBR-Green PCR master mix kit (Roche). The expression of each gene was normalized to the expression of β-actin. The sequences of the primers were: *Rorc*, forward, 5′-CGG AGT TTT CCT TTG GGTG-3′ and reverse, 5′-CCG AGG TAT CTC AGT CAC GAA-3′; *Rora*, forward, 5′-GAA CAC CTT GCC CAG AAC AT-3′ and reverse, 5′-AGC TGC CAC ATC ACC TCT CT-3′; *Il17a*, forward, 5′-CTC ACA CGA GGC ACA AG-3′ and reverse, 5′-CTC AGC AGC AGC AAC AG-3′; *Il17f*, forward, 5′-GGG AAG AAG CAG CCA TTG-3′ and reverse, 5′-TCC AGG GGA GGA CAG TT-3′; *Il21*, forward, 5′-AAA CTC AAG CCA TCA AAC CC-3′ and reverse, 5′-GGT GTC CTT TTC TCA TAC GAA TC-3′; *Il23r*, forward, 5′-GCA GGA AGT ATT TGG TAT GGG -3′ and reverse, 5′-GAA ATG ATG GAC GCA GAA GG-3′; *Actb*, forward, 5′-GCC CCC CTG AAC CCT AA-3′ and reverse, 5′-GAC AGC ACA GCC TGG AT-3′; *Tbx21*, forward, 5′-CGT GGA GGT GAA TGA TGG-3′ and reverse, 5′-GTG ATC TCT GCG TTC TGG TAG-3′; *Ifng*, forward, 5′-ATC AGC AAC AAC ATA AGC GT-3′ and reverse, 5′-GAC CTC AAA CTT GGC AAT ACTC-3′; *Hif1a*, forward, 5′-CGG CGA GAA CGA GAA GAA-3′ and reverse, 5′-GCA ACT GAT GAG CAA GCT CATA-3′.

### Nuclear extract preparation

Cells were harvested and lysed in cytoplasmic extract (CE) buffer (10 mM Tris-HCl PH7.4, 10 mM NaCl and 3 mM MgCl_2_) containing 0.1% (primary T cells) or 0.2% NP-40 (for HeLa cells) for 4 min on ice. Cell lysates were centrifuged at 5,000 r.p.m. for 3 min and the supernatants obtained represented cytoplasmic extracts. Pellets were washed and lysed with RIPA buffer (50 mM Tris-HCl pH 7.4, 1% NP-40, 0.25% Sodium deoxycholate, 150 mM NaCl and 1 mM EDTA). The supernatants obtained after centrifuging at 13,200 r.p.m. for 10 min represented the nuclear extracts.

### Western blot

Cell lysates or immunoprecipitates were resolved by SDS–PAGE and were transferred to PVDF membrane (Millipore) using transfer buffer (30 mM Tris, 250 mM glycine, 1 mM EDTA, 20% methanol) at 4 °C for 2 h at 400 mA. The membrane was treated with block buffer (5% non-fat milk in 0.1% TBST (10 mM Tris-HCl pH 8.0, 150 mM NaCl, 0.1% Tween-20)) at room temperature for 1 h. The membrane was then incubated with primary antibodies overnight at 4 °C, followed by horseradish peroxidase-conjugated secondary antibodies in blocking buffer at room temperature for 1.5 h. The membrane was developed using ECL western blotting detection kit (GE Healthcare), and the chemiluminescence detected by X-ray film. Western blot images have been cropped for presentation. Full size images are presented in Supplementary Figs 12–17.

### Regulatory T cells

nTregs and iTregs (CD4^+^CD25^+^) were purified by FACS sorting. For *in vitro* suppressive assays, CD4^+^CD25^−^ cells (Teff) were placed into round-bottom 96-well plates at 4 × 10^4^ cells per well (for nTreg) or 1 × 10^5^ cells per well (for iTreg), together with anti-CD3 (1 μg ml^−1^), 3 × 10^5^ γ-irradiated autologous presenting cells, and different ratios of Treg cells. Cells were incubated for 72 h, and the incorporation of [^3^H]thymidine was determined.

### Intracellular localization of DAPK and HIF-1α/PHD2

Cells were fixed with 4% (v/v) PFA in PBS, and permeabilized with 0.1% (v/v) Triton X-100. The fixed cells were incubated with blocking buffer (5% BSA in PBS) at room temperature for 1 h, followed by primary antibodies in blocking buffer overnight at 4 °C. The primary antibodies were anti-FLAG (Sigma, 1:400), anti-Myc (Cell Signaling, 1:400), anti-human HIF-1α (BD-Biosciences, 1:100), anti-mouse HIF-1α (Cayman Chemicals, 1:100), anti-DAPK (Sigma, 1:50) and anti-PHD2 (Cell Signaling, 1:50). Cells were washed and incubated with Alexa Flour-labelled secondary antibodies, and were then mounted in DAPI Fluoromount-G buffer (SouthernBiotech, Birmingham, AL, USA). Confocal images were obtained with a Zeiss LSM 780 confocal microscope (Jena, Germany) with an objective lens of Plan-Apochromat 63 × /1.4 Oil DIC M27 at room temperature. DAPI-bound DNA was excited at 405 nm and collected at 420 to 475 nm. Alexa Fluor 488 dye was excited at 488 nm and collected at 500 to 550 nm. Alexa Fluor 555 dye was excited at 555 nm and collected at 568 to 629 nm. The pinhole was at 53 μm, with a 9.8 μm section.

### Retroviral infection

DAPK cDNA was cloned into retroviral vector GFP-RV and then transfected into HEK293T cells. Retroviruses were isolated from the culture supernatants of the transfected HEK293T cells by centrifugation at 26,000 r.p.m. for 2 h at 4 °C on a Beckman SW28 rotor. The viruses were resuspended in PBS containing 5% BSA overnight at 4 °C and stored at −80 °C. Purified WT CD4^+^ T cells were activated with plate-bound anti-CD3 (2 μg ml^−1^) and anti-CD28 (1 μg ml^−1^) with the addition of IL-2 (100 U ml^−1^) for 48 h. Activated T cells were infected with retrovirus in the presence of 8 μg ml^−1^ polybrene by spinning at 2,000 r.p.m. for 1 h at room temperature. Infected T cells were cultured in complete RPMI medium with IL-2 (100 U ml^−1^) for 48 h. GFP^+^ cells were sorted by FACSVantage SE, and were cultured in complete RPMI medium plus IL-2 (100 U ml^−1^) for 2 days before hypoxic treatment.

### *In vitro* binding assays

pGEX-4T3-HIF-1α (GST-HIF-1α) was transformed in BL21 (DE3) *Escherichia coli*, and recombinant GST-HIF-1α fusion proteins purified on a glutathione Sepharose 4B column. RK5F-DAPK (DAPK-FLAG) and pcDNA4-PHD2 (FLAG-PHD2) were transfected into HEK293T cells. Transfected cells were collected and lysed with WCE buffer (25 mM pH 7.7 HEPEs, 300 mM NaCl, 0.1% Triton X-100, 1.5 mM MgCl_2_, 0.2 mM EDTA, 0.1 mM Na_3_VO4, 50 mM NaF and 0.5 mM DTT). Recombinant FLAG fusion proteins were purified with anti-FLAG M2 agarose. Purified recombinant proteins were incubated in PBS at 4 °C for 4 h, and then incubated with anti-GST antibody overnight at 4 °C. Samples were incubated with protein G magnetic beads (GE) at 4 °C for 1 h. The immune complexes were washed with WCE buffer and examined by western blot.

### DAPK kinase assay

DAPK kinase assays were performed as previously described^62^. DAPK proteins were isolated by anti-FLAG from SF21 insect cells and incubated with 5 μg GST-HIF-1α or GST-MLC in kinase reaction buffer (50 mM HEPES pH 7.5, 8 mM MgCl_2_, 2 mM MnCl_2_, 0.5 mM CaCl_2_, 0.1 mg ml^−1^ bovine serum albumin (BSA), 1 μM bovine CaM (Sigma), 50 μM ATP, 10 μCi [γ-32P]ATP) at 25 °C for 15 min. The reactions were stopped by adding SDS sample buffer, and resolved on SDS–PAGE. The phosphorylation was detected by autoradiography.

### Statistics

Our data were randomly collected but were not blinded. We did not exclude any data in this study. Our data mostly meet the assumption of the tests (normal distribution). Microsoft Office Excel and Prism 5.0 (GraphPad software) were used for data analysis. Unpaired two-tailed Student *t*-tests were used to compare results from between two groups. Data were presented as mean with standard deviation (s.d.) or standard error of the mean (s.e.m.), as indicated in the figure legend.

### Data availability

All relevant data are available from the authors upon request.

## Additional information

**How to cite this article:** Chou, T.-F. *et al.* Tumour suppressor death-associated protein kinase targets cytoplasmic HIF-1α for Th17 suppression. *Nat. Commun.* 7:11904 doi: 10.1038/ncomms11904 (2016).

## Supplementary Material

Supplementary Information Supplementary Figures 1-17
