# Supplementary material for: Tumour suppressor death-associated protein kinase targets cytoplasmic HIF-1α for Th17 suppression
Source: Nat Commun. 2016 Jun 17;7:11904. doi: 10.1038/ncomms11904 (PMC4915028; doi:10.1038/ncomms11904)
Supplement: Supplementary Information — Supplementary Figures 1-17 [file ncomms11904-s1.pdf]

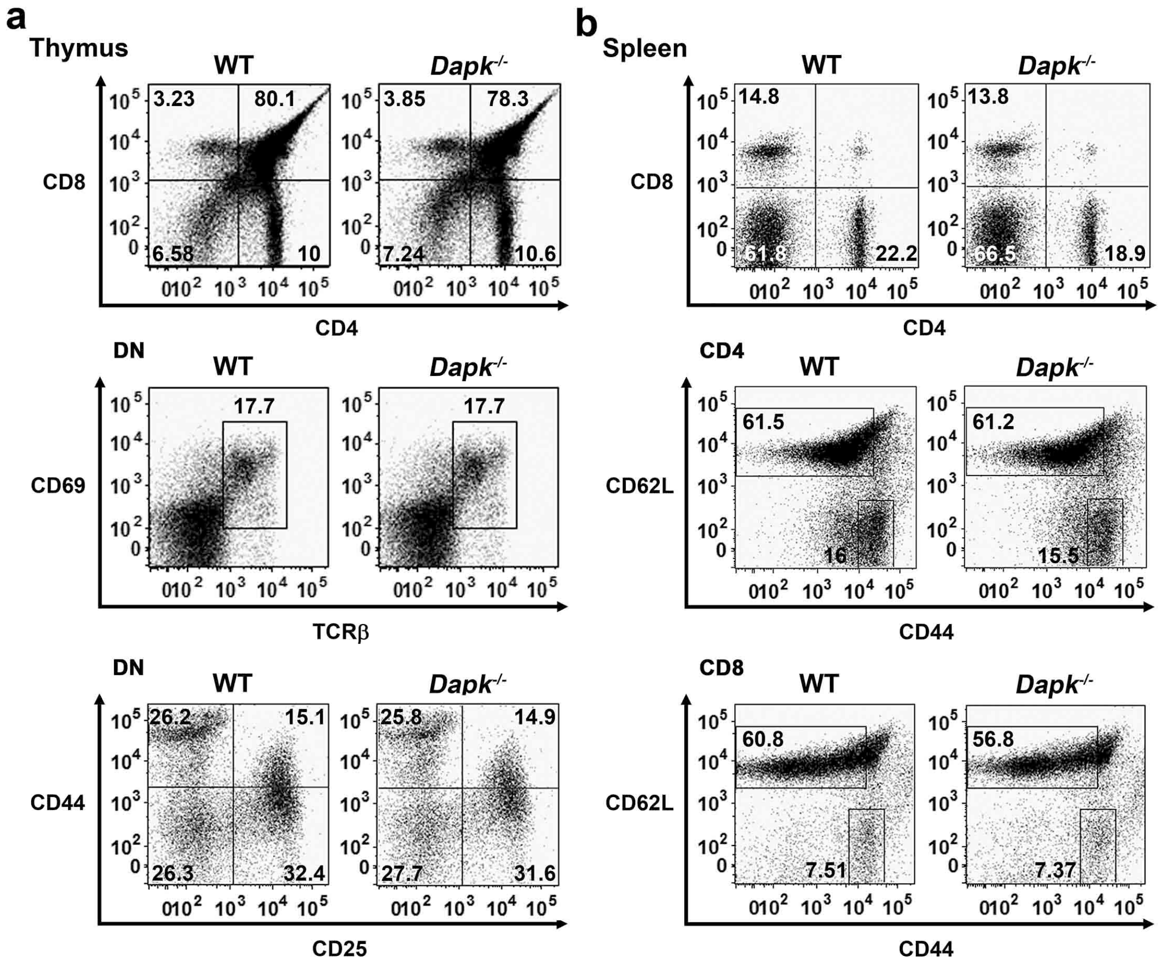

Supplementary Figure 1. Normal T lymphocyte populations in *Dapk*$^{-/-}$ mice. (**a**) Normal thymic development in *Dapk*$^{-/-}$ mice. Thymocytes from WT and *Dapk*$^{-/-}$ mice were stained for expression of CD4 and CD8. Numbers indicate percentages of each fraction. Lower panels: the $CD4^{-}CD8^{-}$ double negative (DN) thymocytes were gated and analyzed for the expression of CD69, TCRβ, CD25 and CD44. (**b**) Normal peripheral T cell population in *Dapk*$^{-/-}$ mice. The frequencies of splenic $CD4^{+}$ and $CD8^{+}$ T cells from WT and *Dapk*$^{-/-}$ mice, and the fractions of naïve ($CD44^{-}CD62L^{+}$) and memory T cells from WT and *Dapk*$^{-/-}$ $CD4^{+}$ and $CD8^{+}$ T cells were determined by flow cytometry. Data are representative of three mice.

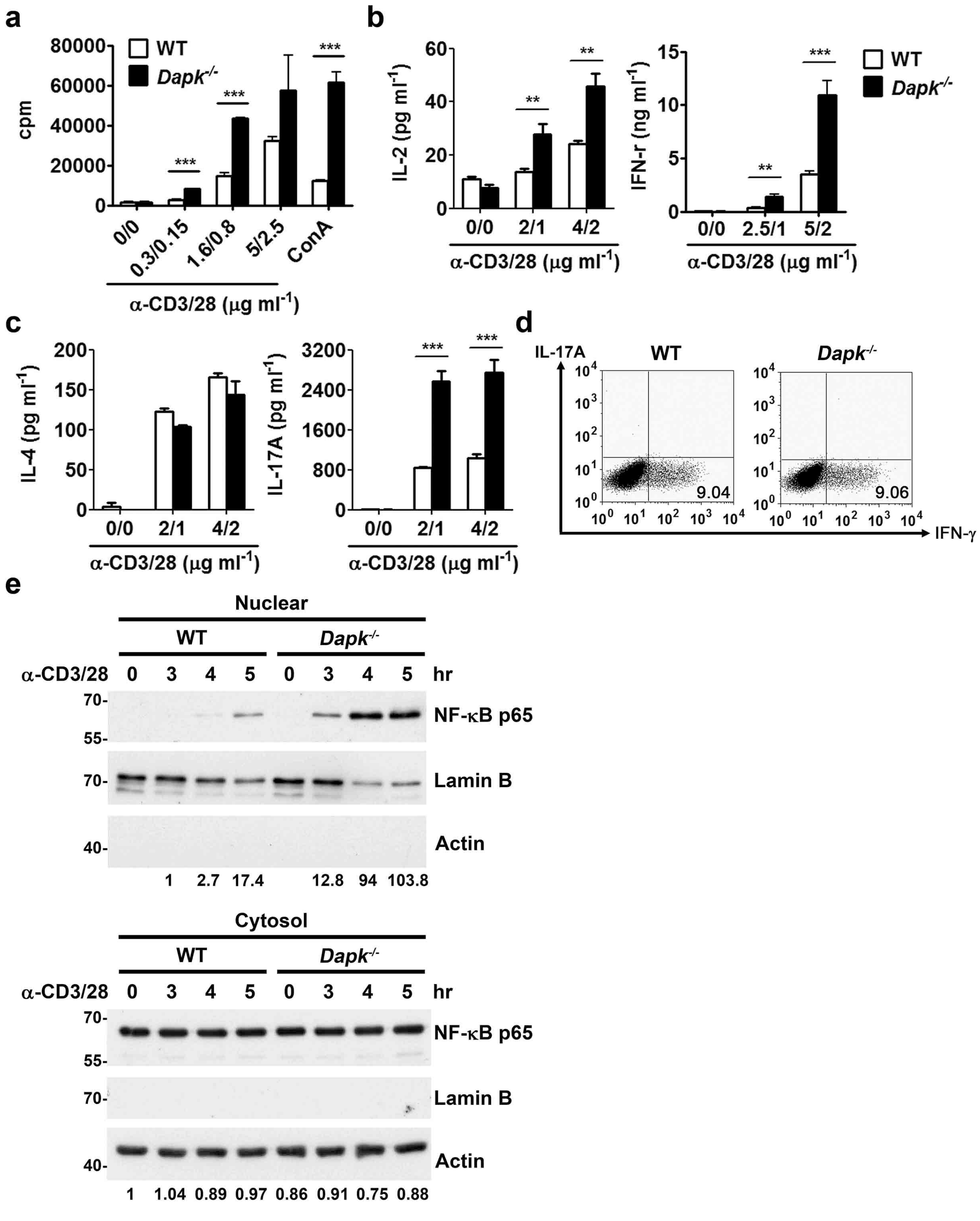

Supplementary Figure 2. Enhanced activation in *Dapk*$^{-/-}$ T cells. (**a**) Increased proliferation in *Dapk*$^{-/-}$ T cells. Purified WT and *Dapk*$^{-/-}$ splenic T cells were stimulated with plate-bound anti-CD3 (5 μg ml^{-1}) and anti-CD28 (2.5 μg ml^{-1}) for 60 hr, and proliferation was determined by incorporation of [^{3}H]thymidine. (**b, c**) Increased selective cytokine production in *Dapk*$^{-/-}$ T cells. Total splenic T cells from WT and *Dapk*$^{-/-}$ mice were stimulated with anti-CD3/CD28, and the secreted IL-2 and IFN-γ were determined at 24 hr and 48 hr, respectively (b). Activated T cells were treated with IL-2

for 2 days, followed by CD3/CD28 restimulation for 48 hr, and the production of IL-4 and IL-17 quantitated (c). Values (a-c) are mean ± s.d., n=3. ***P* < 0.01, ****P* < 0.001 for unpaired t-test (a-c). (**d**) Normal IFN-γ production in restimulated *Dapk*$^{-/-}$ T cells. Activated T cells were treated with IL-2 for 3 days, followed by TPA/A23187 restimulation, and the expression of IFN-γ analyzed by intracellular staining and flow cytometry. (**e**) Enhanced NF-κB activation in *Dapk*$^{-/-}$ T cells. T cells from WT and *Dapk*$^{-/-}$ mice were stimulated with anti-CD3/CD28, and the cytosolic and nuclear extracts were collected at the indicated time points. The cytosolic and nuclear contents of NF-κB p65 were analyzed by Western blot. Lamin B and actin was used as markers for the nucleus and cytoplasm, respectively. For quantitation, p65 levels were normalized against lamin B or actin. The normalized nuclear p65 level in WT T cells 3 h after activation is set at 1, while normalized cytosolic p65 content in resting WT T cells is set at 1. Data (a-e) are representative of three independent experiments.

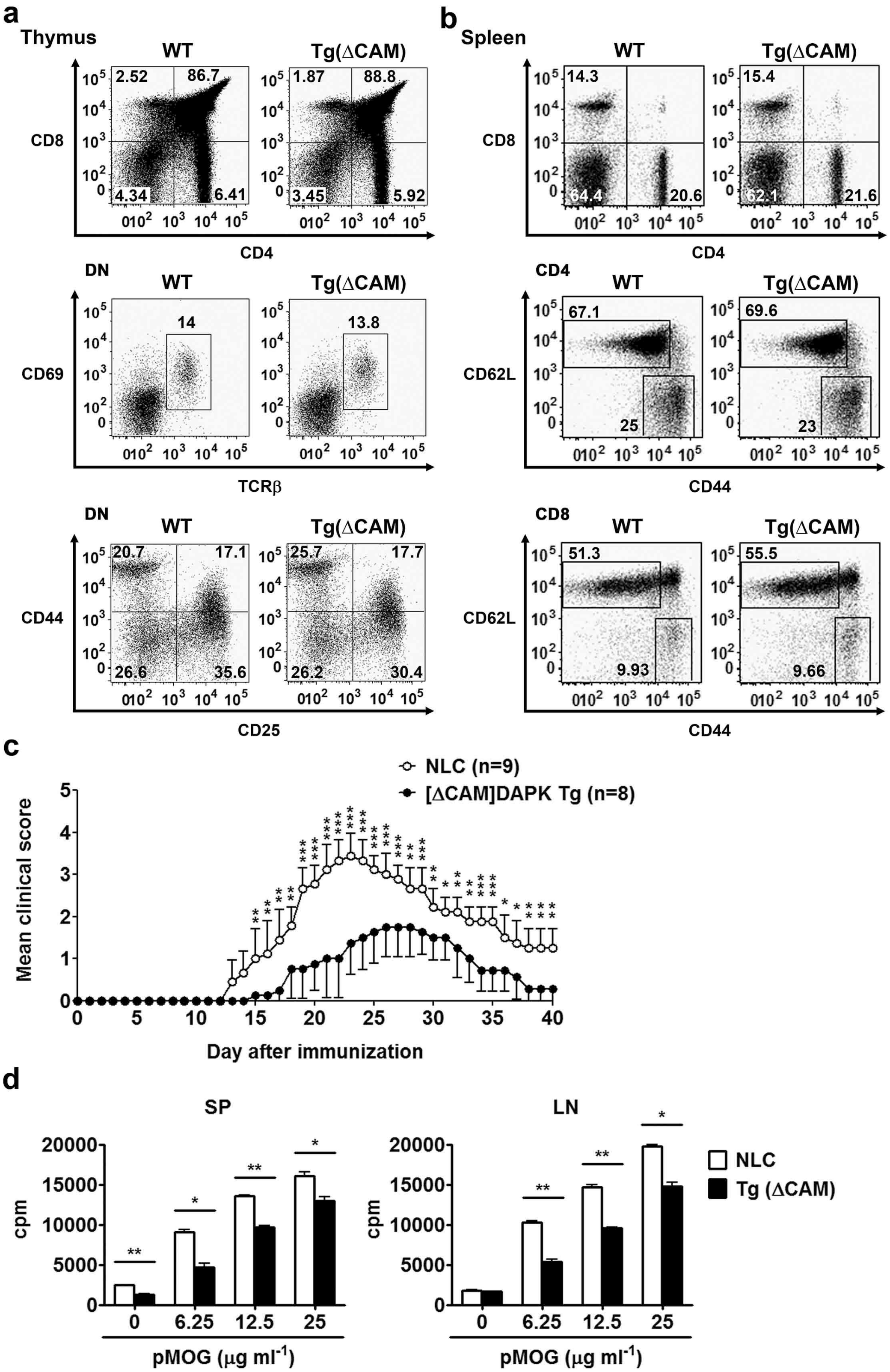

Supplementary Figure 3. Attenuated EAE in *Dapk*-transgenic mice. (a) Normal thymic development in [ΔCAM]DAPK-transgenic mice. Thymocytes from WT and

[ΔCAM]DAPK-transgenic mice were stained for expression of CD4 and CD8. Numbers indicate percentages of each fraction. Lower panels: the $CD4^{-}CD8^{-}$ double negative (DN) thymocytes were gated and analyzed for the expression of CD69, TCRβ, CD25 and CD44. (**b**) Normal peripheral T cell population in [ΔCAM]DAPK-transgenic mice. The frequency of splenic $CD4^{+}$ and $CD8^{+}$ T cells from WT and [ΔCAM]DAPK-transgenic mice, and the fraction of naïve ($CD44^{-}CD62L^{+}$) and memory T cells from WT and [ΔCAM]DAPK-transgenic $CD4^{+}$ and $CD8^{+}$ T cells were determined by flow cytometry. Data (a, b) are representative of three mice. (**c**) T cell-specific [ΔCAM]DAPK suppressed EAE. [ΔCAM]DAPK-transgenic and normal littermate control (NLC) mice were immunized with 400 μg MOG (33-55), and the disease progression of EAE was measured. Values are mean ± s.d. $*P < 0.05$, $**P < 0.01$, $***P < 0.001$ for unpaired t-test. (**d**) Reduced response to MOG (33-55) in [ΔCAM]DAPK-transgenic T cells. Splenic (SP) and lymph node (LN) $CD4^{+}$ T cells were isolated from [ΔCAM]DAPK-transgenic and NLC mice 25 days after immunization. Cells were stimulated with irradiated autologous presenting cells and MOG peptide, and T cell proliferation was determined 72 h later by incorporation of ^{3}H-thymidine. Values are mean ± s.d., n=3. $*P < 0.05$, $**P < 0.01$ for unpaired t-test. Data are representative of three independent experiments.

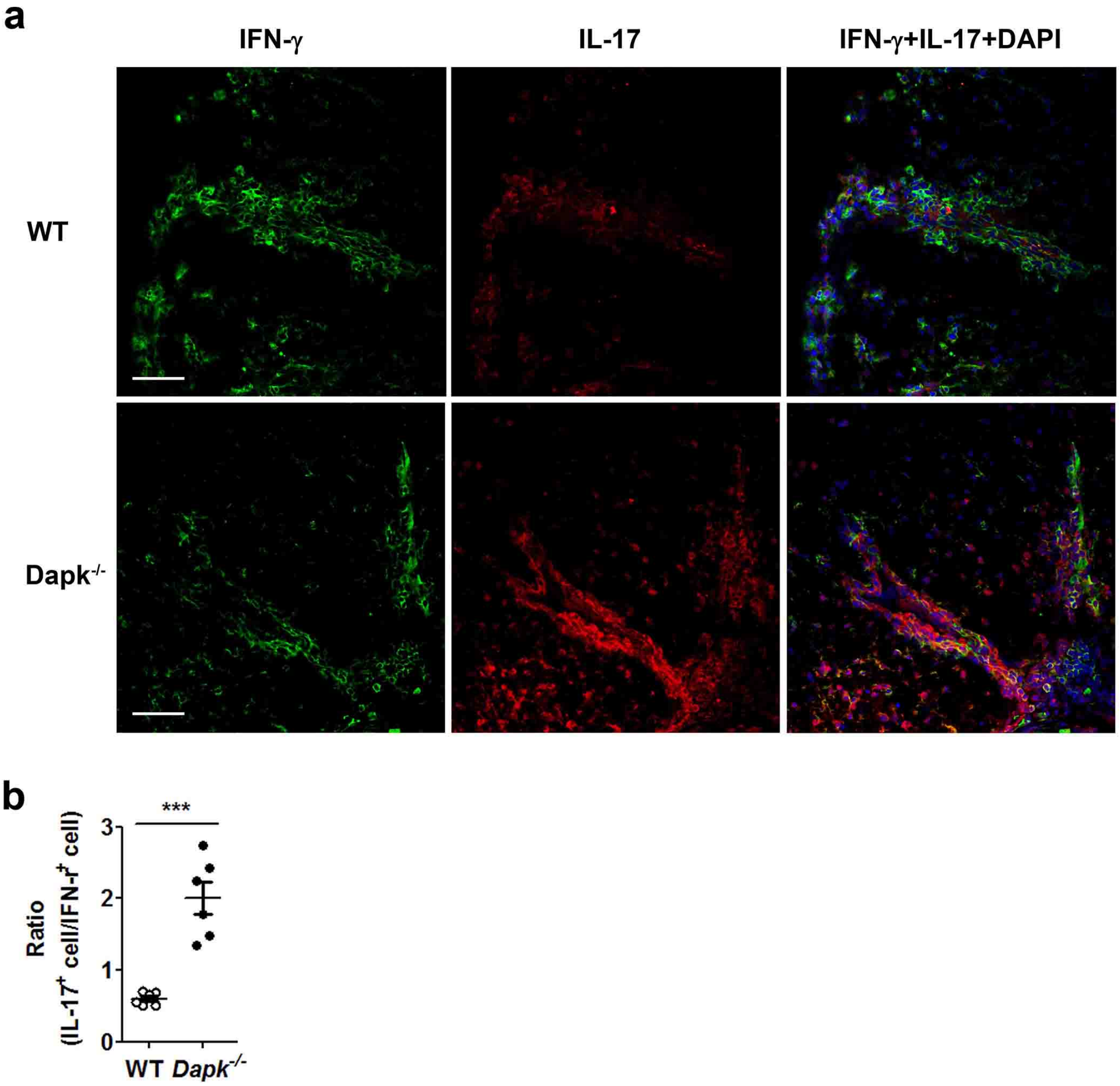

Supplementary Figure 4. Increased IL-17-expressing mononuclear cells in spinal cords from *Dapk*$^{-/-}$ mice sensitized for EAE generation. (**a**) Staining of the infiltrated IFN-γ^+ cells and IL-17$^+$ cells from spinal cords of mice at peak encephalomyelitis. Spinal cords from WT and *Dapk*$^{-/-}$ mice were isolated 18 days and 15 days after MOG immunization, respectively. Spinal cords were fixed and frozen sections obtained. Tissue sections were stained with anti-IFN-γ (green), anti-IL-17 (red), and DAPI, and analyzed by confocal microscopy. Scale bar, 50 μm. (**b**) Quantitation of results from (a). The ratio of IL-17$^+$ cells to IFN-γ^+ cells in spinal cord were calculated based on six measurements obtained from three pairs of WT and *Dapk*$^{-/-}$ mice induced with EAE. Values are mean ± s.e.m. ***$P < 0.001$ for unpaired t-test.

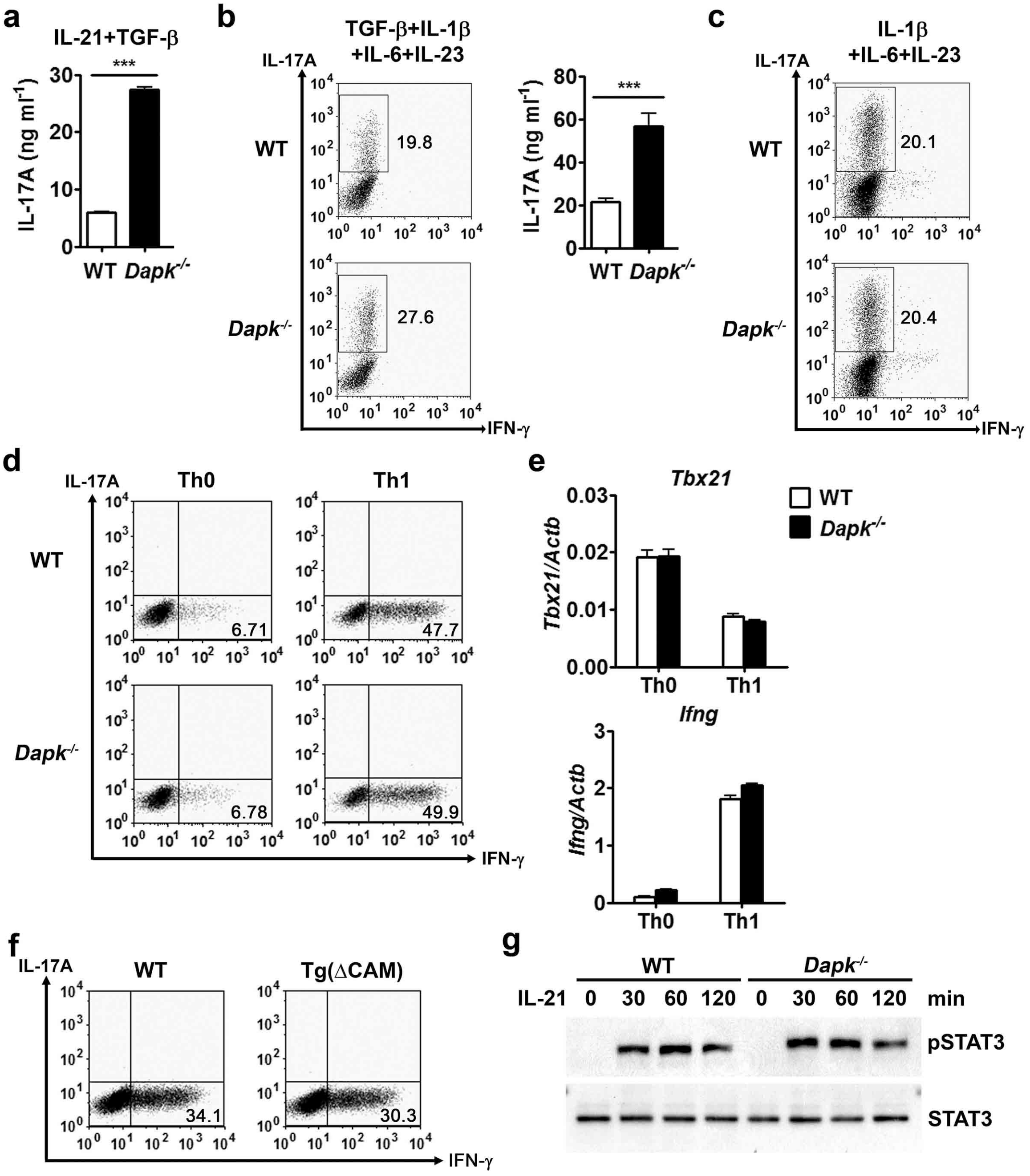

Supplementary Figure 5. Increased Th17 generation and normal Th1 differentiation in *Dapk$^{-/-}$* T cells. (**a**) Enhanced IL-17 secretion by *Dapk$^{-/-}$* Th17 cells induced with IL-21 and TGF-β. Th17 cells were generated by incubation of naïve T cells with IL-21 and TGF-β for 5 days. The production of IL-17 by WT and *Dapk$^{-/-}$* Th17 cells after restimulation with TPA/A23187 was measured by ELISA. Values are mean ± s.d., n=3. ***$P < 0.001$ for unpaired t-test. (**b**) Increased IL-17 expression by *Dapk$^{-/-}$* Th17 cells differentiated with TGF-β, IL-1β, IL-6 and IL-23. WT and *Dapk$^{-/-}$* naïve CD4 T cells were differentiated into Th17 cells for 3 days in the presence of TGF-β (2 ng ml^{-1}), IL-1β (50 pg ml^{-1}), IL-6 (1 ng ml^{-1}), and IL-23 (1 ng ml^{-1}), and the expression of IL-17 determined by intracellular staining (left panel) and ELISA (right panel). Values are

mean ± s.d., n=3. ***$P < 0.001$ for unpaired t-test. (**c**) No effect of DAPK-deficiency on Th17 cells differentiated with IL-1β, IL-6 and IL-23. WT and *Dapk*$^{-/-}$ naïve CD4 T cells were differentiated into Th17 cells for 3 days in the presence of IL-1β (20 ng ml^{-1}), IL-6 (20 ng ml^{-1}) and IL-23 (20 ng ml^{-1}), and the expression of IL-17 determined. (**d**) Th1 development is not affected by DAPK-deficiency. Naïve CD4 T cells from control and *Dapk*$^{-/-}$ mice were subjected to differentiation into Th0 and Th1 cells for 3 days. The generation of IFN-γ and IL-17 after TPA/A23187 restimulation was determined by intracellular staining. (**e**) Normal induction of *Ifng* and *Tbx21* in *Dapk*$^{-/-}$ T cells. WT and *Dapk*$^{-/-}$ Th0 and Th1 cells were restimulated with TPA/A23187 for 3 h, and RNA was isolated. The expression of *Ifng* and *Tbx21* were determined by quantitative PCR. Values are mean ± s.d., n=2. (**f**) [ΔCAM]DAPK transgene does not affect Th1 development. Naïve CD4 T cells from control and [ΔCAM]DAPK-transgenic mice were differentiated into Th1 cells for 3 days. The generation of IFN-γ and IL-17 after restimulation was determined by intracellular staining. (**g**) Normal IL-21-induced STAT3 phosphorylation in *Dapk*$^{-/-}$ T cells. Freshly isolated WT and *Dapk*$^{-/-}$ T cells were stimulated with IL-21 for the indicated times. The levels of phospho-STAT3 and total STAT3 were examined by Western blotting. Data are representative of three (a, d, e, f) or two (b, c, g) independent experiments.

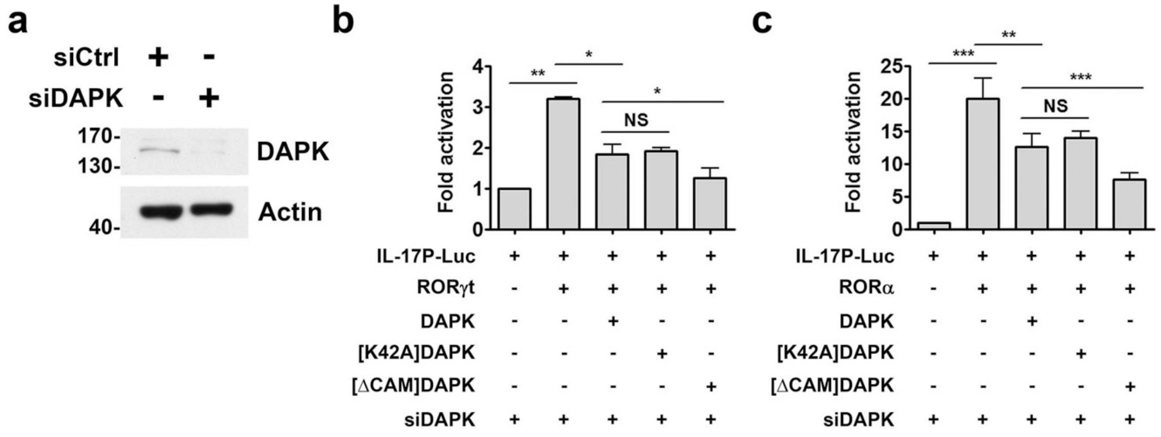

Supplementary Figure 6. DAPK also inhibits the capacity of RORγt and RORα to activate the IL-17 promoter. (**a**) Knockdown of DAPK in 293T cells. 293T cells were transfected with siRNA or DAPK-specific siRNA, and the levels of DAPK determined 48 h later. (**b, c**) DAPK affects RORγt- and RORα-directed IL-17 promoter activation. 293T cells were transfected with DAPK-specific siRNA. Then, 24 h later, IL-17P-Luc, DAPK, [K42A]DAPK, [ΔCAM]DAPK, RORγt (b), or RORα (c) was transfected into DAPK-knockdown 293T cells as indicated. Luciferase activities were determined after another 24 h. Values (b, c) are mean ± s.d., n=3. *$P < 0.05$, **$P < 0.01$, ***$P < 0.001$ for unpaired t-test. Data (a-c) are representative of three independent experiments.

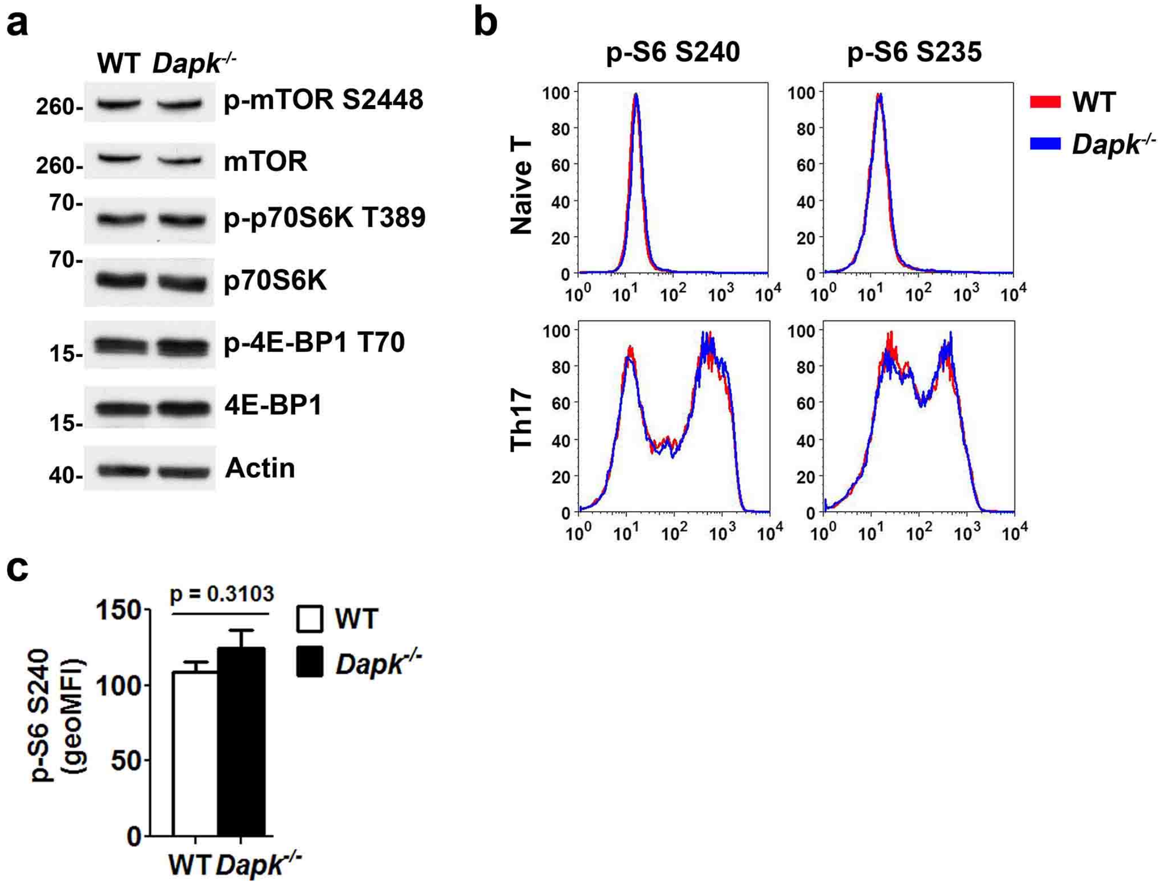

Supplementary Figure 7. DAPK-deficiency does not affect mTOR signaling. (**a**) Normal mTORC1 signaling in *Dapk*$^{-/-}$ T cells. Naïve T cells from WT and *Dapk*$^{-/-}$ mice were differentiated under Th17 conditions for 24 hr. Cell lysates were prepared and phospho-mTOR (Ser2448), phospho-p70S6K (Thr389), phospho-4E-BP1 (Thr70), mTOR, p70S6K, and 4E-BP1 levels were examined by Western blot. (**b, c**) Normal ribosome S6 phosphorylation in *Dapk*$^{-/-}$ T cells. The phosphorylation of ribosome S6 at S235/236 and S240/244 was determined by flow cytometry in WT and *Dapk*$^{-/-}$ T cells one day after differentiation into Th17 cells (b). The geoMFI of three independent experiments is calculated (c).

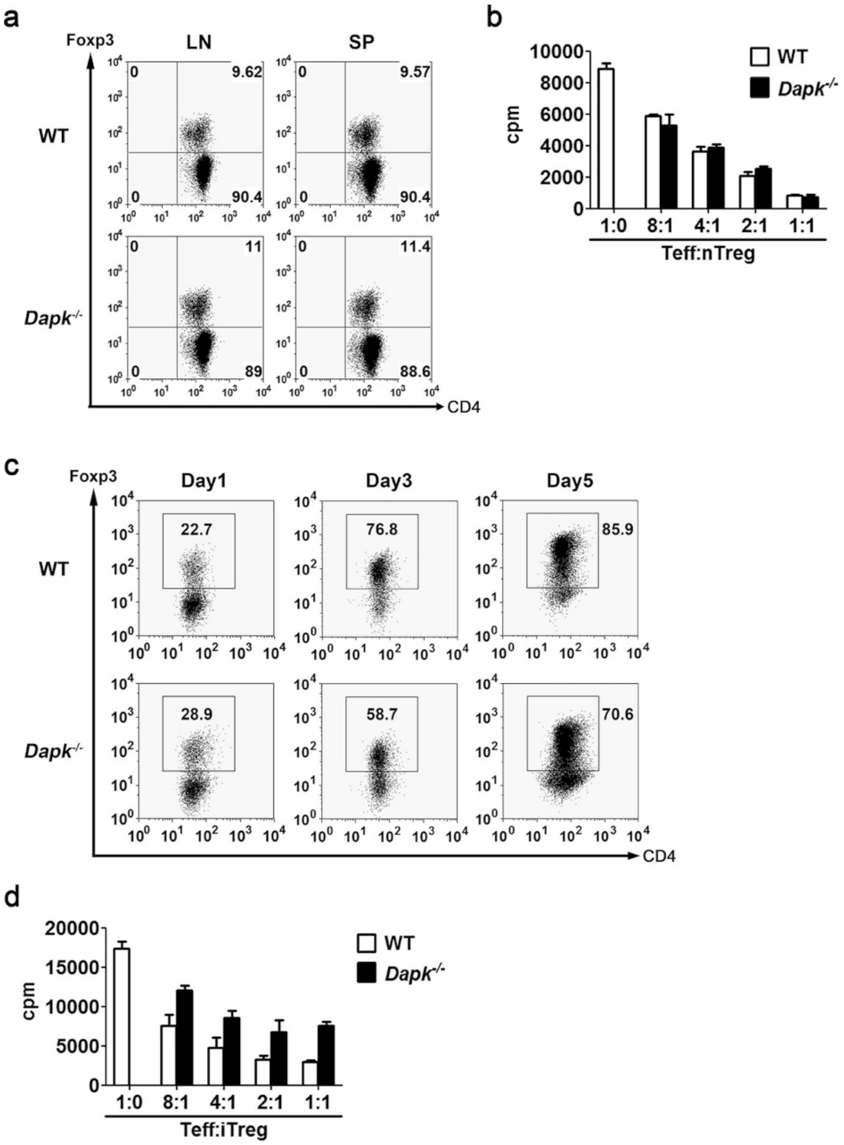

Supplementary Figure 8. DAPK-deficiency impairs induced regulatory T cell function. (**a**) Normal nTreg population in *Dapk*$^{-/-}$ mice. Lymph node and splenic T cells were isolated from WT and *Dapk*$^{-/-}$ mice, and the CD4^{+}Foxp3^{+} population determined by flow cytometry. (**b**) Normal *in vitro* suppressive activity of *Dapk*$^{-/-}$ nTregs. WT and *Dapk*$^{-/-}$ nTreg (CD4^{+}CD25^{+}) cells were isolated and co-cultured with γ-irradiated autologous presenting cells and indicated amounts of WT effector T cells (Teff, CD4^{+}CD25^{-}) plus soluble anti-CD3 for 72 hr. Proliferation was determined by incorporation of ^{3}H-thymidine. Mean ± s.d., n=4. (**c**) Attenuated iTreg differentiation from *Dapk*$^{-/-}$ T cells. Purified CD4^{+}CD25^{-} T cells were stimulated with plate-bound anti-CD3 (5 μg ml^{-1}) and anti-CD28 (1 μg ml^{-1}) in the presence of TGF-β (5 ng ml^{-1}) and IL-2 (20 ng ml^{-1}) for 1-5 days. The expression of Foxp3 was determined at 1, 3 and 5 days after treatment. (**d**) Impaired suppressive activity of *Dapk*$^{-/-}$ iTreg cells. The *in vitro* suppression activity of iTregs at day 5 (c) was analyzed. Mean ± s.d., n=3. Data are representative of three (a, b) and two (c, d) independent experiments.

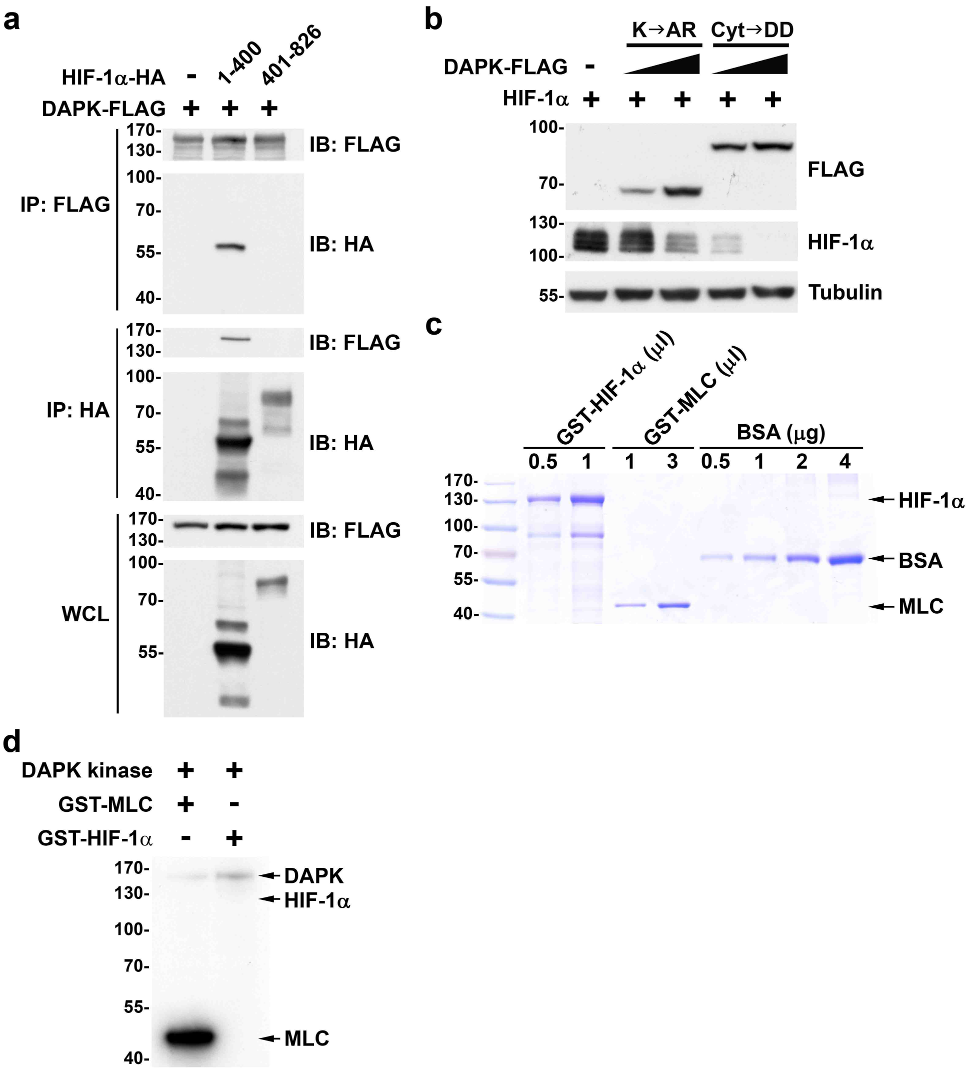

Supplementary Figure 9. DAPK interacts with HIF-1α but the kinase domain of DAPK is not essential for HIF-1α down-regulation. (**a**) DAPK binds the N-terminus of HIF-1α. HEK293T cells were transfected with DAPK-FLAG and the N-terminus (amino acids 1-400) or C-terminus (amino acids 401-826) of HIF-1α-HA. Cell lysates were immunoprecipitated with anti-FLAG (top) or anti-HA (bottom), and the contents of HIF-1α and DAPK were examined by Western blot. (**b**) DAPK fragment containing the kinase domain was ineffective in inducing HIF-1α downregulation. HEK293T cells were transfected with HIF-1α and the FLAG-tagged N-terminal (kinase to ankyrin repeats, K→AR) or C-terminal (Cytoskeleton to death domain, Cyt→DD) of DAPK. Forty-eight hr after transfection, the expression of HIF-1α was determined by immunoblots. (**c**) Expression of recombinant HIF-1α. Recombinant GST-HIF-1α and GST-MLC was purified from *E. coli*, and the purity assessed by Coomassie blue staining. (**d**) HIF-1α is not phosphorylated by DAPK *in vitro*. Recombinant DAPK was incubated with GST-HIF-1α or GST-MLC in kinase buffer containing [γ-^{32}P]ATP at 25°C for 15 min. The reaction mixtures were resolved by SDS-PAGE, and the phosphorylation of MLC (positive control) and HIF-1α examined by autoradiography. Data (a-d) are representative of two independent experiments.

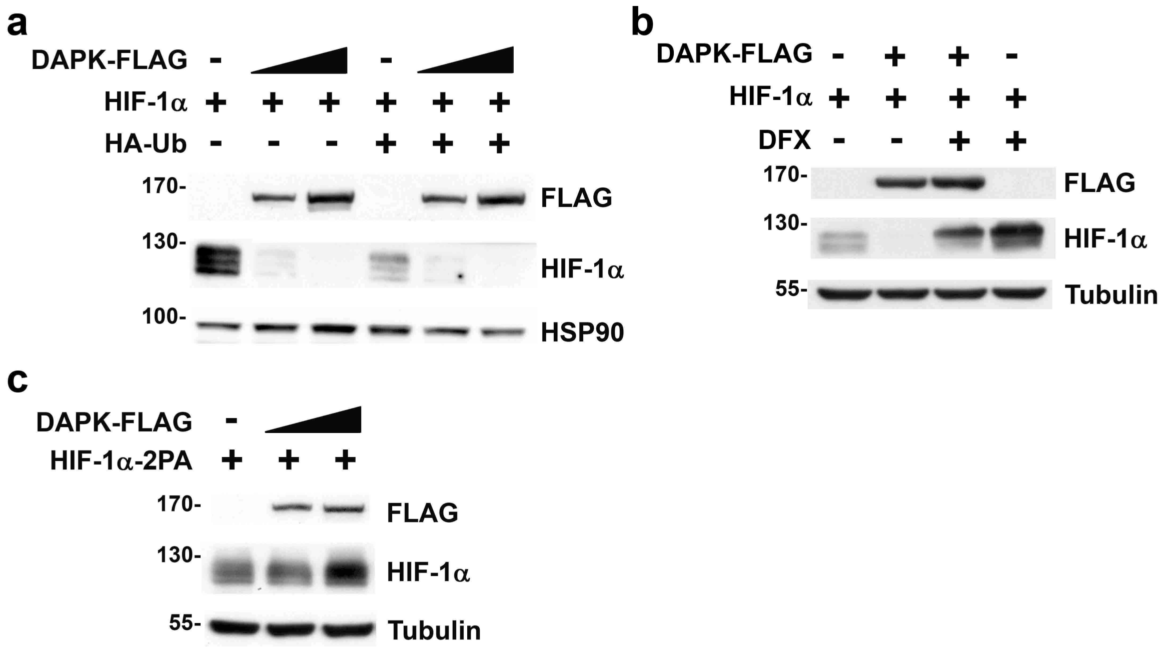

Supplementary Figure 10. Inhibition of PHD prevents DAPK-mediated HIF-1α degradation. (**a**) Ubiquitin enhances DAPK-triggered HIF-1α down-regulation. HEK-293T cells were transfected with DAPK-FLAG, HIF-lα and HA-ubiquitin, and the levels of DAPK-FLAG and HIF-1α were determined 48 h after transfection. (**b**) PHD inhibitor prevents DAPK-induced HIF-1α degradation. HEK293T cells were transfected with DAPK and HIF-1α and treated with or without deferoxamine mesylate (DFX, 100 μM) 32 h after transfection. Cells were harvested 16 h after DFX addition, and the contents of HIF-1α were determined. (**c**) Mutation of proline hydroxylation sites confers resistance of HIF-1α to DAPK-induced degradation. HEK293T cells were transfected with DAPK-FLAG and HIF-1α[P402A/P564A] (HIF-1α-2PA), and the contents of HIF-1α were determined 48 h after transfection. Data (a-c) are representative of three independent experiments.

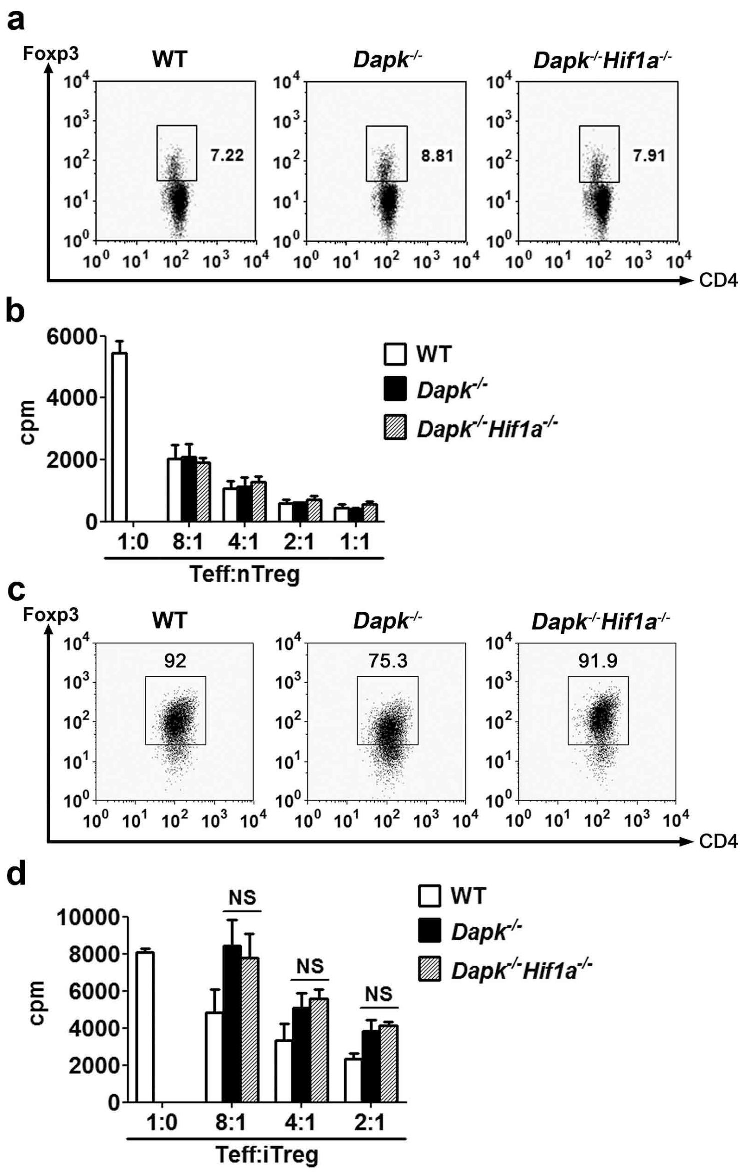

Supplementary Figure 11. Additional knockout of HIF-1α does not restore defects of ***Dapk*$^{-/-}$ iTregs.** (**a**) Normal Treg population in *Dapk*$^{-/-}$*Hif1a*$^{-/-}$ mice. Lymph node and splenic T cells were isolated from WT, *Dapk*$^{-/-}$ or *Dapk*$^{-/-}$*Hif1a*$^{-/-}$ mice, and the $CD4^+Foxp3^+$ population was determined by flow cytometry. (**b**) Normal *in vitro* suppressive activity of *Dapk*$^{-/-}$*Hif1a*$^{-/-}$ nTregs. WT, *Dapk*$^{-/-}$ or *Dapk*$^{-/-}$*Hif1a*$^{-/-}$ nTreg cells were isolated and co-cultured with irradiated WT presenting cells, indicated ratios of WT effector T cells and anti-CD3 for 72 hr before proliferation was determined. (**c**) HIF-1α-deficiency restores *Dapk*$^{-/-}$ iTreg differentiation. Purified $CD4^+CD25^-$ T cells from WT, *Dapk*$^{-/-}$ and *Dapk*$^{-/-}$*Hif1a*$^{-/-}$ mice were stimulated with anti-CD3/anti-CD28 in the presence of TGF-β and IL-2 for 5 days. The expression of Foxp3 was determined. (**d**) Impaired suppressive activity of *Dapk*$^{-/-}$ iTreg cells is not corrected by additional HIF-1α-knockout. The *in vitro* suppression activity of iTregs at day 5 (c) was analyzed. Values (b, d) are mean ± s.d., n=3. NS, not significant. Data (a-d) are representative of three independent experiments.

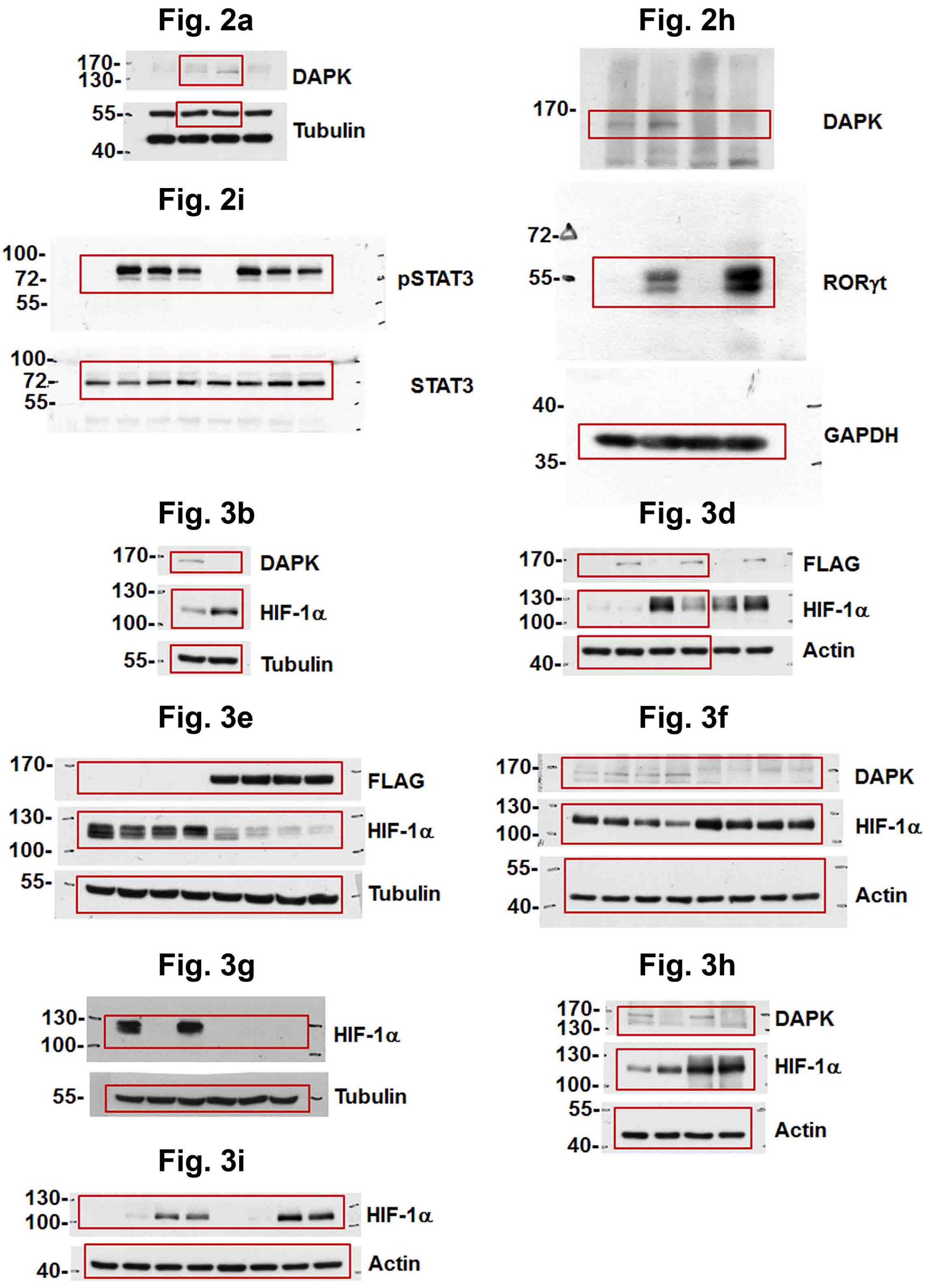

Supplementary Figure 12. Uncropped images of the original scans of immunoblots. Uncropped, full-size scans of immunoblots shown in Fig. 2a, 2h, 2i, 3b, and 3d-3i.

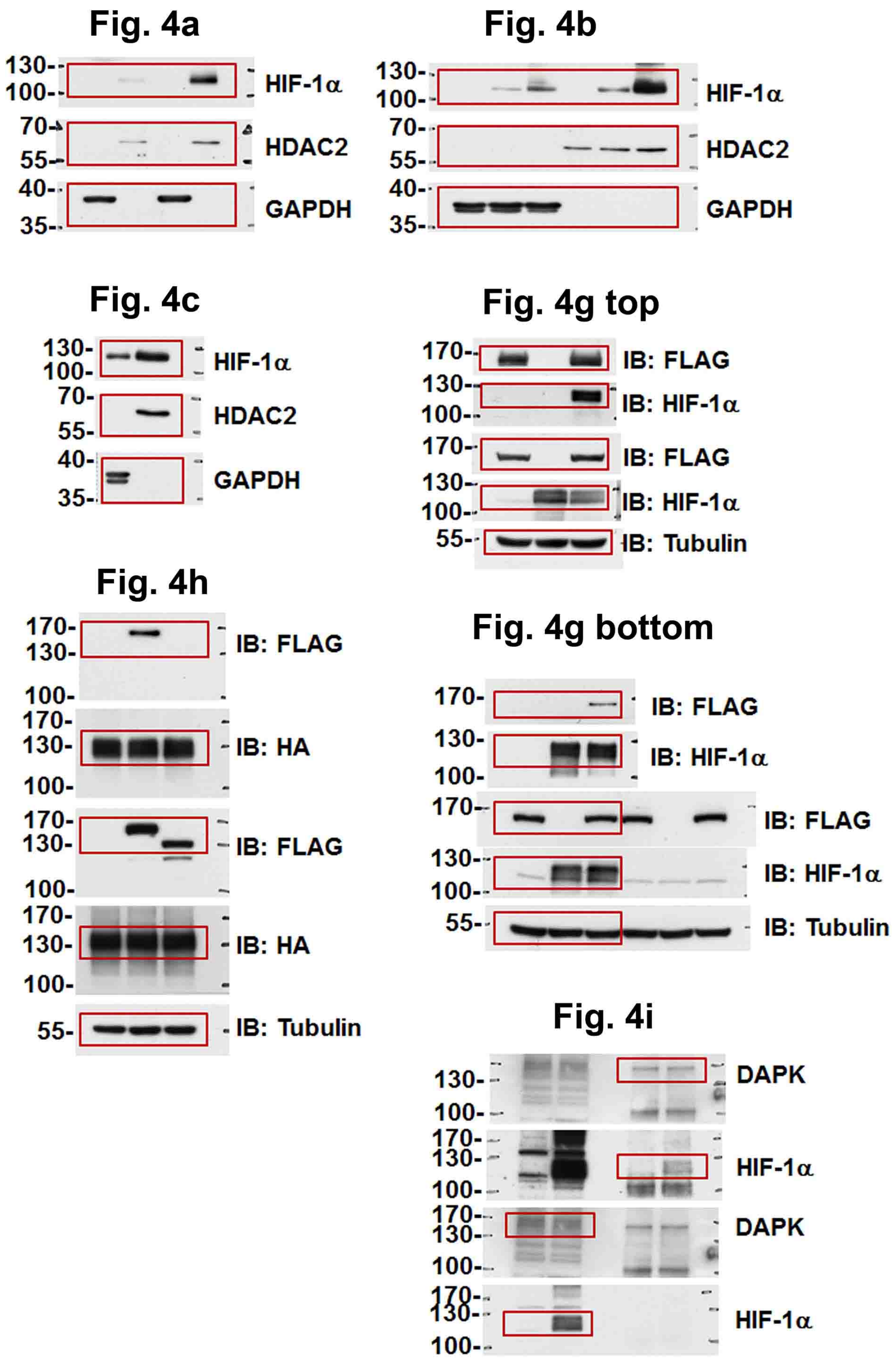

Supplementary Figure 13. Uncropped images of the original scans of immunoblots. Uncropped, full-size scans of immunoblots shown in Fig. 4a-4c and 4g-4i.

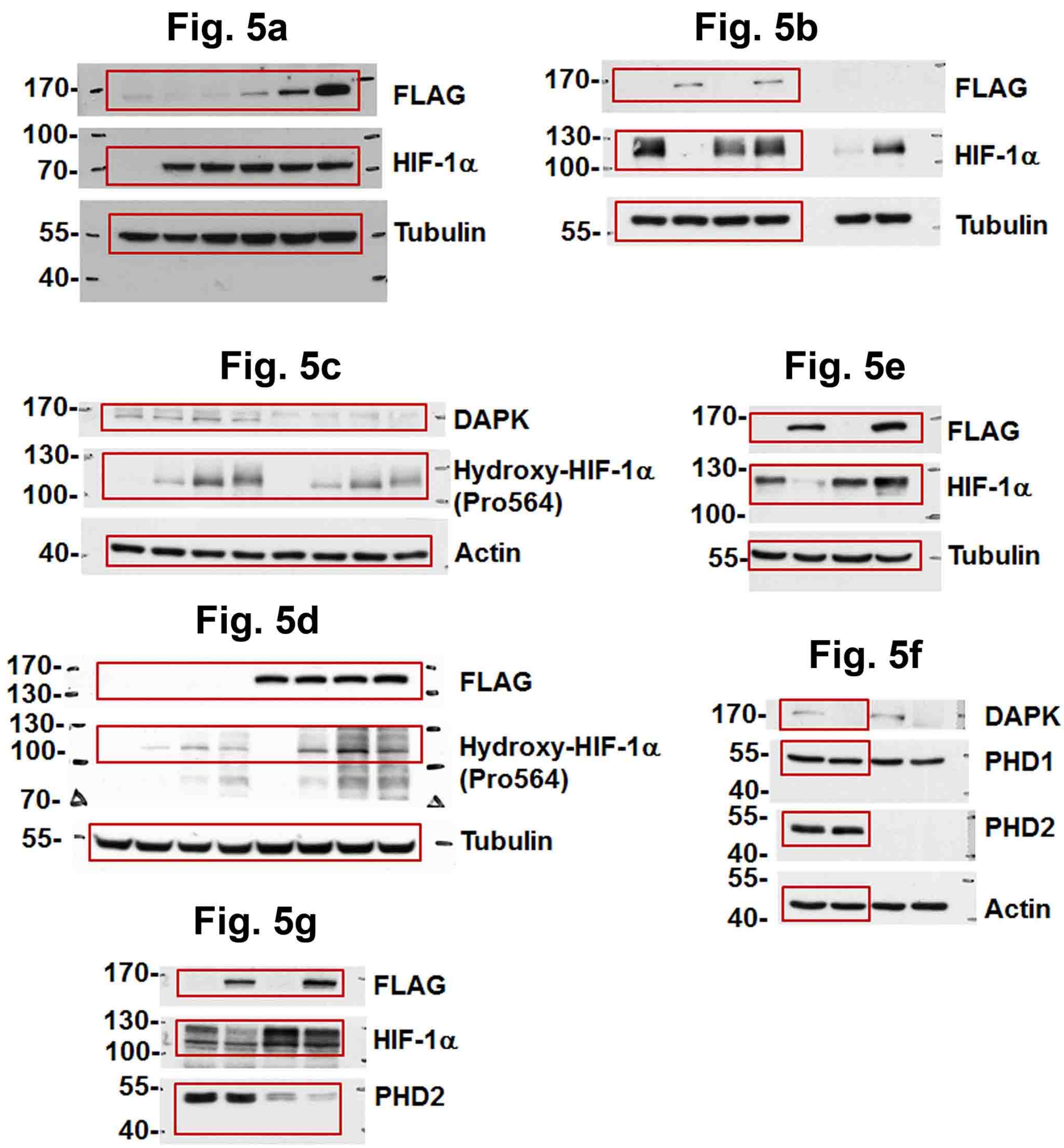

Supplementary Figure 14. Uncropped images of the original scans of immunoblots. Uncropped, full-size scans of immunoblots shown in Fig. 5a -5g.

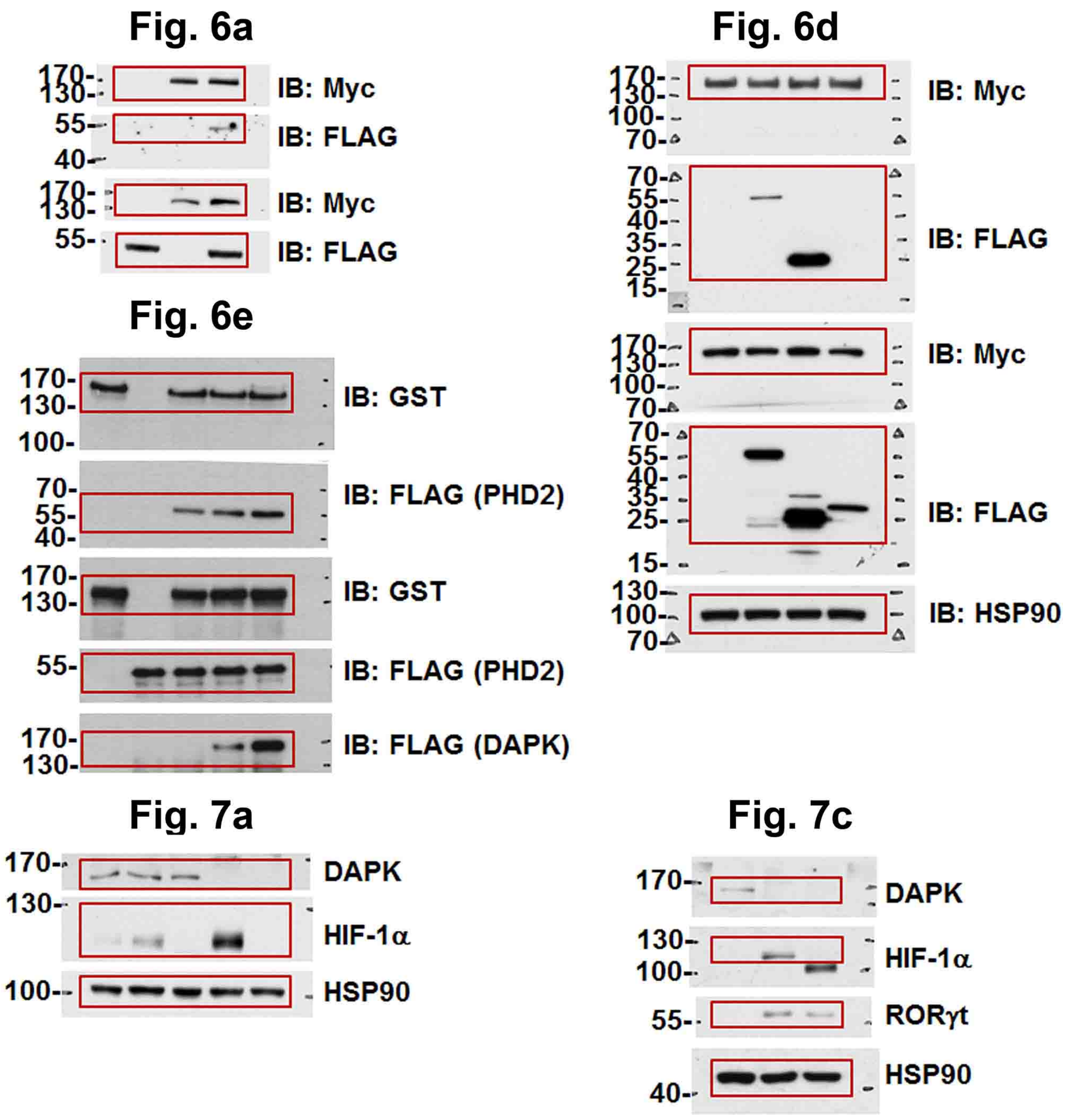

Supplementary Figure 15. Uncropped images of the original scans of immunoblots. Uncropped, full-size scans of immunoblots shown in Fig. 6a, 6d, 6e, 7a, and 7c.

Supplementary Fig. 2e

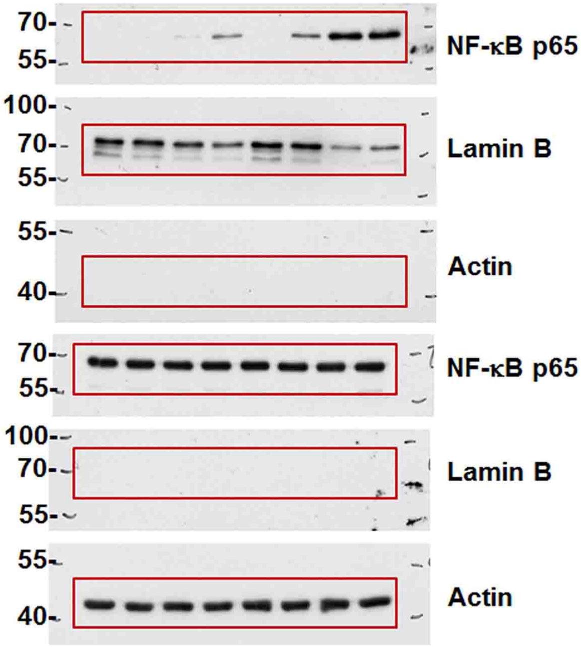

Supplementary Fig. 5g

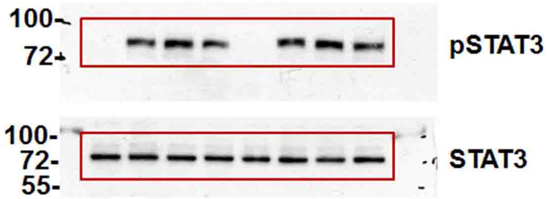

Supplementary Fig. 6a

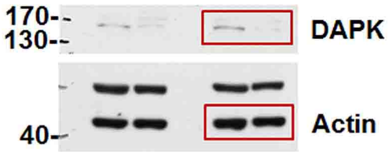

Supplementary Fig. 7a

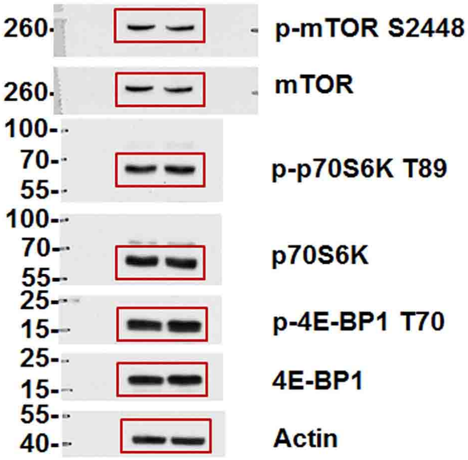

Supplementary Fig. 9a

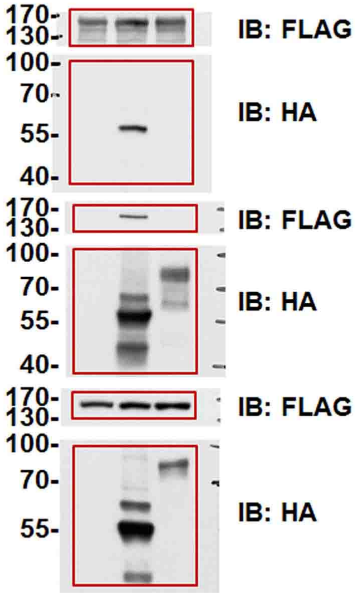

Supplementary Figure 16. Uncropped images of the original scans of immunoblots. Uncropped, full-size scans of immunoblots shown in Supplementary Fig. 2e, 5g, 6a, 7a, and 9a.

Supplementary Fig. 9b

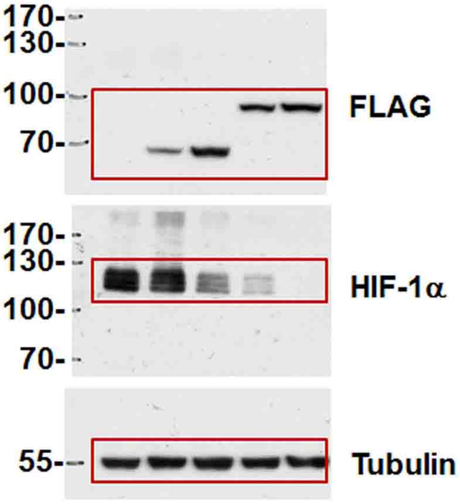

Supplementary Fig. 9c

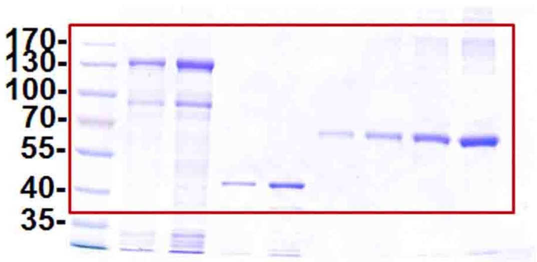

Supplementary Fig. 9d

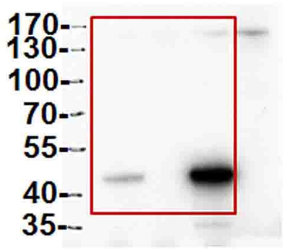

Supplementary Fig. 10a

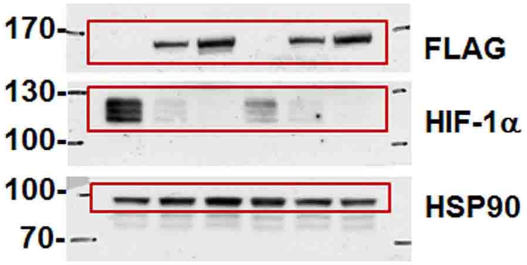

Supplementary Fig. 10b

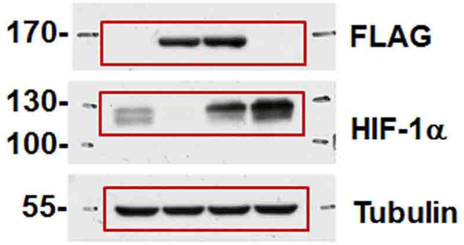

Supplementary Fig. 10c

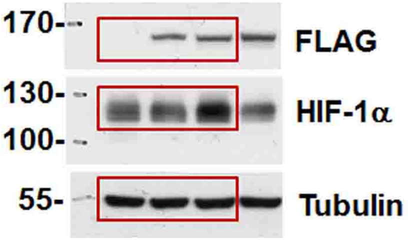

Supplementary Figure 17. Uncropped images of the original scans of immunoblots. Uncropped, full-size scans of immunoblots shown in Supplementary Fig. 9b-9d and 10a-10c.
